# *MtNRLK1*, a CLAVATA1-like leucine-rich repeat receptor-like kinase upregulated during nodulation in *Medicago truncatula*

**DOI:** 10.1038/s41598-018-20359-4

**Published:** 2018-02-01

**Authors:** Carole Laffont, Carolien De Cuyper, Justine Fromentin, Virginie Mortier, Annick De Keyser, Christa Verplancke, Marcelle Holsters, Sofie Goormachtig, Florian Frugier

**Affiliations:** 1 Institute of Plant Sciences—Paris Saclay (IPS2), CNRS, INRA, U Paris-Sud, U Paris-Diderot, U d’Evry, Université Paris-Saclay, Bâtiment 630, 91190 Gif-sur-Yvette, France; 2 Department Plant Biotechnology and Bioinformatics, Ghent University, 9052 Ghent, Belgium; 3 Department of Plant Systems Biology, VIB, 9052 Ghent, Belgium

## Abstract

Peptides are signaling molecules regulating various aspects of plant development, including the balance between cell division and differentiation in different meristems. Among those, CLAVATA3/Embryo Surrounding Region-related (CLE-ESR) peptide activity depends on leucine-rich-repeat receptor-like-kinases (LRR-RLK) belonging to the subclass XI. In legume plants, such as the *Medicago truncatula* model, specific CLE peptides were shown to regulate root symbiotic nodulation depending on the LRR-RLK SUNN (Super Numeric Nodules). Amongst the ten *M. truncatula* LRR-RLK most closely related to SUNN, only one showed a nodule-induced expression, and was so-called MtNRLK1 (Nodule-induced Receptor-Like Kinase 1). *MtNRLK1* expression is associated to root and nodule vasculature as well as to the proximal meristem and rhizobial infection zone in the nodule apex. Except for the root vasculature, the *MtNRLK1* symbiotic expression pattern is different than the one of *MtSUNN*. Functional analyses either based on RNA interference, insertional mutagenesis, and overexpression of *MtNRLK1* however failed to identify a significant nodulation phenotype, either regarding the number, size, organization or nitrogen fixation capacity of the symbiotic organs formed.

## Introduction

Plant growth relies on stem cells located in meristems from which most tissues and organs are post-embryonically derived e.g. the Shoot and Root Apical Meristems (SAM and RAM)^1–3^. Plants tightly control the balance between cell division and differentiation in order to maintain a specific number of dividing stem cells to sustain indeterminate growth and at the same time to provide enough cells for differentiation into appropriate tissues and organs^2,4,5^. In Arabidopsis, the CLAVATA3 (CLV3) peptide controls stem cell maintenance and organ specification in the SAM^6–8^. These CLV3 peptides are secreted from the outer cell layers of the SAM and act on the central domain depending on a variety of homo- and heteromultimeric complexes of plasma membrane-localized Leucine-Rich Repeats Receptor-Like Kinases (LRR-RLKs), including CLV1, BAM1 (BARELY ANY MERISTEM 1), BAM2, BAM3, CLV2, CRN (CORYNE) and RPK2 (RECEPTOR-LIKE PROTEIN KINASE 2)^9^ (and references therein). In the RAM of Arabidopsis, an analogous pathway fine tunes the balance between stem cell differentiation and maintenance^10^. CLE40 would act through the CLV1 and ARABIDOPSIS CRINKLY 4 (ACR4) receptors to regulate the Quiescent Center stem cell niche^10,11^.

When legume plants are deprived of nitrogen, nodule organs form on their root systems following a symbiotic interaction with nitrogen-fixing soil bacteria, collectively named rhizobia. The specific recognition between the two symbiotic partners allows bacteria to penetrate root hairs and progress towards the root cortex within infection threads^12^. Meanwhile, cell divisions are activated in cortical and pericycle cells^13^, leading to the formation of a nodule primordium that is colonized by infection threads containing rhizobia. The primordium then differentiates into a nodule in which bacteria fix atmospheric nitrogen into ammonium within the central nitrogen fixation zone, which is then assimilated by plant cells. In *Medicago truncatula* nodules, the apical meristem is continuously acting, leading to an indeterminate growth of the nodule, and is contiguous to a differentiation zone where rhizobial infections occur. The organization of the Nodule Apical Meristem (NAM) is therefore very similar to the RAM and SAM^13–15^, pointing out that a CLE/LRR-RLK may be involved in controlling the balance between cell division and differentiation in the nodule apex. Interestingly, MtCLE12 and MtCLE13 peptides were reported to be expressed in the apical region of the nodule^16^.

As nodulation and nitrogen fixation are energy-consuming processes, a tight negative control of nodule number and activity is exerted by the plant depending on soil nitrogen availability and on the plant photosynthetic capacity to provide carbon required for ammonium assimilation, so-called “Autoregulation of Nodulation” (AON)^17^. Understanding the regulatory mechanisms that allow legume plants to adapt nodulation and nitrogen fixation rates to the balance between fluctuating nitrogen resources, in time and space, and the plant growth energy demand, may open perspectives to optimize plant productivity of legume crops. Such applied objective is key to further reduce nitrogen fertilizer inputs and their associated economic costs and environmental drawbacks, while maintaining, or even improving, legume yields under sustainable agricultural practices. The AON regulatory pathway involves a long-distance root-to-shoot-to-root systemic signaling allowing suppressing subsequent nodule initiation depending on soil nitrogen availability or nodule number. In different legumes including *M. truncatula*, the AON pathway was shown to rely on a CLE/LRR-RLK regulatory module, involving the production in rhizobia inoculated roots of peptides such as MtCLE12/MtCLE13 or LjCLE-RS1/LjCLE-RS2 (CLE-Root Signal) that are proposed to move to shoots through the xylem vasculature and to act depending on the MtSUNN (Super Numeric Nodules)/LjHAR1 (Hypernodulation and Aberrant Root)/GmNARK (Nodule Autoregulation Receptor Kinase) subclass XI LRR-RLK receptor closely related to Arabidopsis CLV1^16–25^. In Arabidopsis, CLE6 overexpression in roots affects shoot morphology^26^, indicating that CLE peptide-mediated long-distance signaling is not specific to legumes. Unlike the Arabidopsis *clv1* mutant however, legume mutants affecting the protein most closely related to AtCLV1 do not show any SAM defect, suggesting a functional diversification among plants and/or different functional redundancies between LRR-RLKs. Other symbiotic AON mutants affected in LRR-RLKs were additionally identified, including CLV2-homologs from pea and Lotus, LjKLAVIER (KLV) that is closely related to the Arabidopsis RPK2 receptor, as well as the MtCRN kinase closely related to AtCRN^18,27,28^, suggesting that, similarly as for SAM and RAM, complexes of LRR-RLK are involved in the AON systemic signaling. Accordingly, MtSUNN was shown to form homo- and hetero-dimers with MtCLV2 and MtCRN^28^. In contrast to the *nark/sunn/har1* AtCLV1-related mutants, MtCLV2- and MtRPK2-related mutants show SAM defects, indicating that partially overlapping receptor complexes controls SAM activity and nodule number in legumes^27,29^.

More recently, another family of secreted peptides, referred to as CEPs (C-terminal Encoding Peptides), mostly produced under nitrogen-starvation, was shown to positively regulate nodulation and to inhibit lateral root formation^30^. Interestingly, opposite phenotypes were observed in the *cra2* (*compact root architecture 2*) mutant which is affected in another subclass XI LRR-RLK^31^. In *Arabidopsis*, CEP peptides regulate root development depending on CEP receptors (CEPR1 and CEPR2) closely related to CRA2^32–34^. Accordingly in *M. truncatula*, the CEP1 peptide requires CRA2 to regulate nodule and lateral root development^35^.

The objective of this work was to identify SUNN-related *M. truncatula* receptors that are associated to the symbiotic nodulation based on their expression pattern. A single gene, *MtNRLK1*, was identified within the 10 proteins most closely related to SUNN as being upregulated by rhizobia. Interestingly, whereas the expression pattern of *MtNRLK1* and *MtSUNN* is overlapping in tissues associated to the root vasculature, *MtNRLK1* expression in the nodule apical region is, in contrast to *MtSUNN*, partially overlapping with the expression patterns of *MtCLE12* and *MtCLE13*, leading to the hypothesis that a putative short-distance action of these peptides in the NAM might rely on the MtNRLK1 receptor. However, MtNRLK1 knock-down and ectopic expression did not reveal any significant nodulation phenotype, either considering nodule number, size, organization, nitrogen fixation capacity, or a requirement for the CLE long distance AON regulatory pathway.

## Results

### Identification of a *MtSUNN*-related LRR-RLK showing an enhanced expression in symbiotic nodules, *MtNRLK1*

The full amino acid sequence of MtSUNN was blasted against the predicted proteome of *M. truncatula* (BlastP against the Mt4.0 database, John Craig Venter Institute [JCVI], http://jcvi.org/medicago/). For nine of the ten best hits, the expression pattern was available at the Medicago Gene Expression Atlas (*Mt*GEA) (http://mtgea.noble.org/v3/) and amongst these, only one gene (*Medtr5g090100*, *M. truncatula* genome v4.0), showed a nodulation-enhanced expression profile and was therefore designated *MtNRLK1* (for Nodule-induced RLK1).

**Figure 1:**
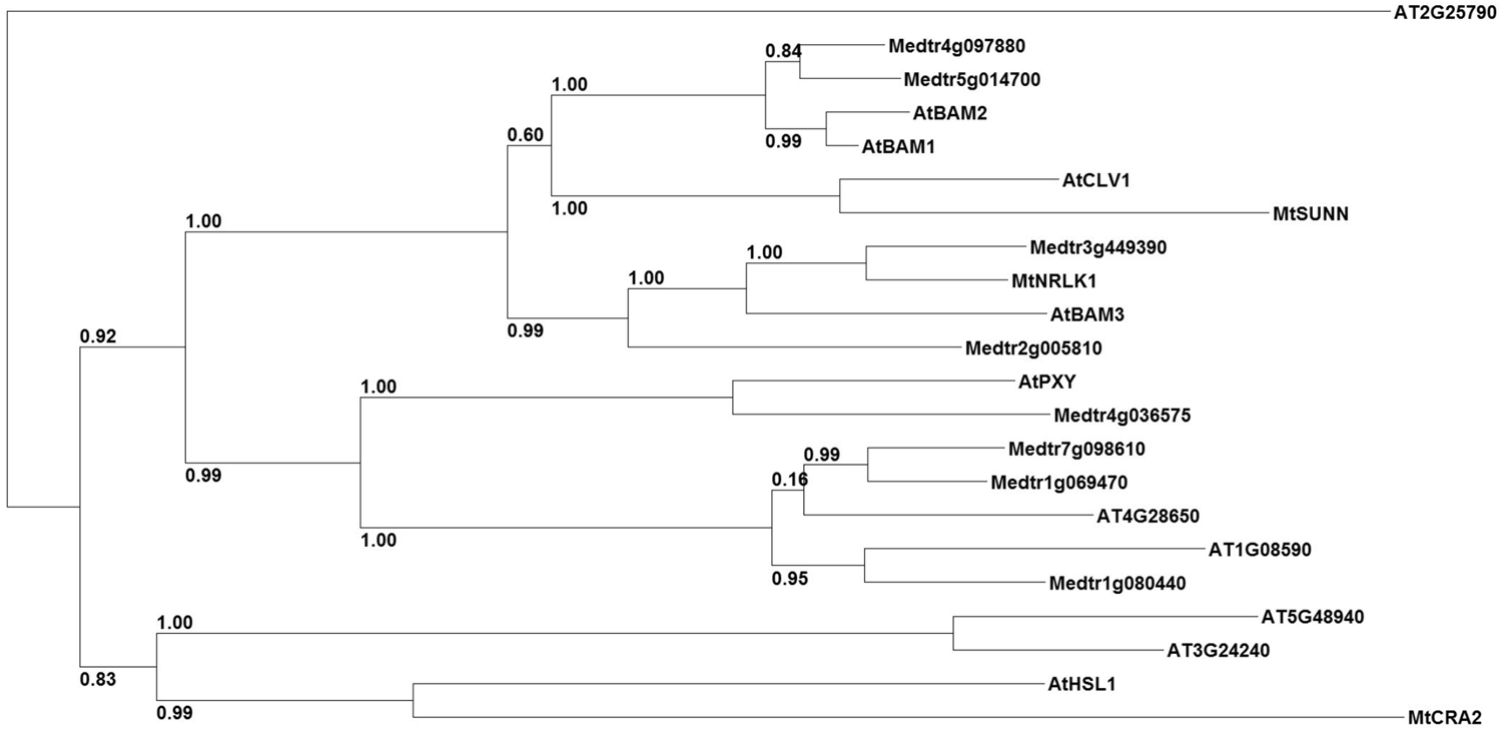
Protein similarity tree of the *M. truncatula* LRR-RLKs most closely related to MtSUNN and of the *A. thaliana* proteins most closely related to MtNRLK1. The tree was designed based on the full amino acid sequences of the ten proteins most closely related to MtSUNN in *M. truncatula*, including MtNRLK1, and of the ten *A. thaliana* proteins most closely related to MtNRLK1. MtCRA2, a LRR-RLK functionally characterized in *M. truncatula* symbiotic nodulation, was also included in the similarity tree. aLRT branch support values are indicated, and considered significant when higher than 0.90.

A similarity tree was designed using MtNRLK1 and the ten most closely related proteins from Arabidopsis and *M. truncatula* (Fig. 1), which all belonged to the subclass XI. The MtCRA2 RLK was also included in the tree, revealing that even though belonging also to the same subclass, it grouped clearly in a different branch of the similarity tree. In Arabidopsis, MtNRLK1 is most closely related to AtBAM3, and to a lower extent with AtCLV1, AtBAM1, AtBAM2 (Figs 1 and S1). Among *M. truncatula* proteins, MtNRLK1 is most closely related to Medtr3g449390 (*M. truncatula* genome v4.0), showing 82% of similarity and 73% of identity. Based on available RNAseq transcriptomic data^36^, the *Medtr3g449390* gene is expressed more strongly in roots than in nodules (Fig. S2), indicating that its symbiotic expression pattern diverges from the one of the closely related *MtNRLK1* gene.

**Figure 2:**
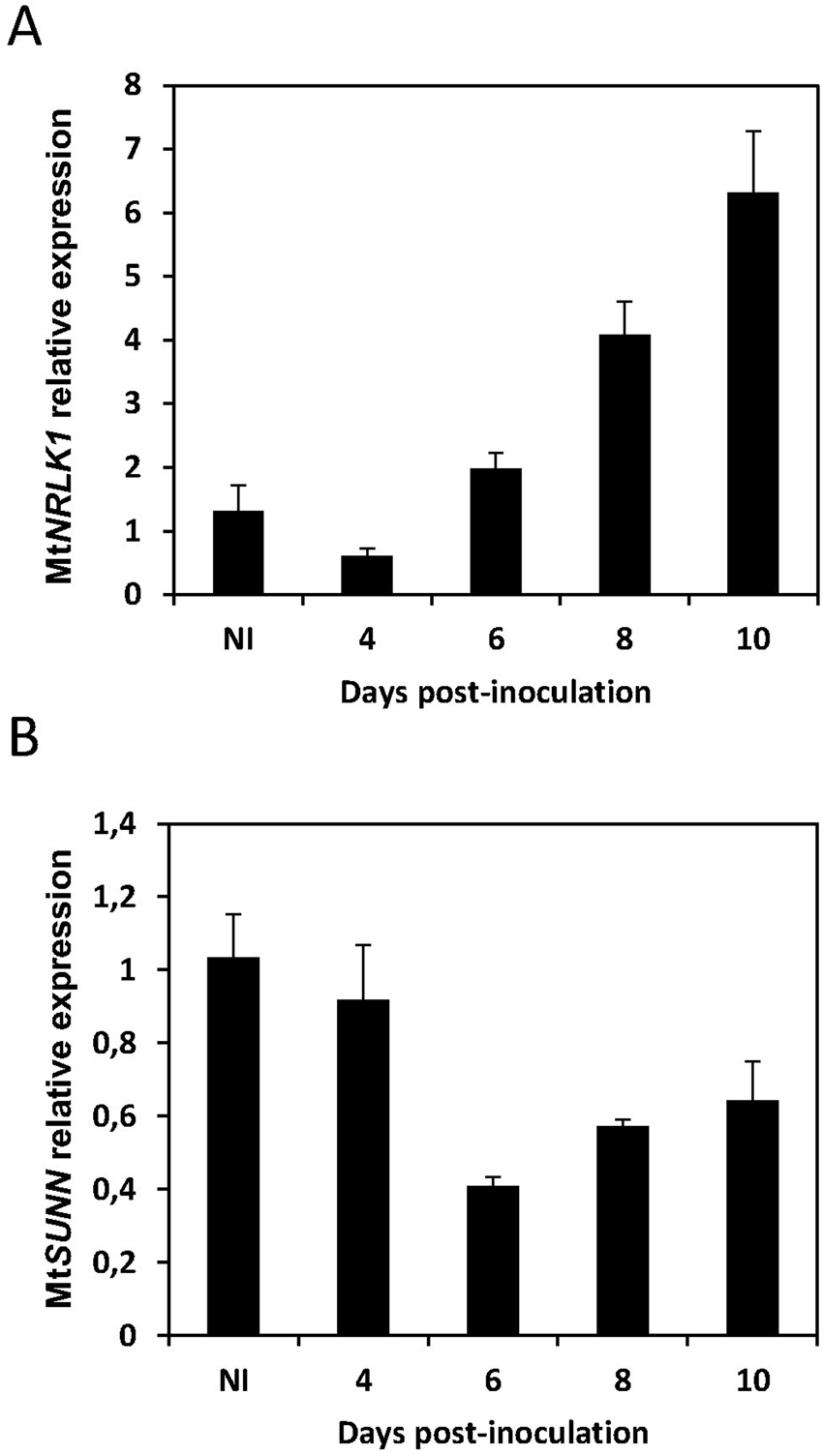
Expression analysis of *MtNRLK1* and *MtSUNN* during a wild-type nodulation kinetic. *MtNRLK1* (**A**) and *MtSUNN* (**B**) expression was analyzed by real-time RT-PCR in susceptible zones of Non-Inoculated roots (NI) or in nodulated roots at 4, 6, 8, and 10 days post-inoculation with *S. meliloti* (dpi). The relative expression of the genes of interest was normalized against the 40S Ribosomal S8 Protein (TC100533, *M. truncatula* Gene Index version 8) reference gene. Two biological replicates are averaged and error bars represent Standard Deviation (SD).

To refine the analysis of *MtNRLK1* expression during nodule development, relative transcript levels were determined by real-time Reverse-Transcriptase PCR (qRT-PCR) in a *Sinorhizobium meliloti*-induced nodulation kinetic (Fig. 2A). The differentiation zone of non-inoculated (NI) roots, corresponding to the optimal nodulation susceptible zone, was used as a reference. *MtNRLK1* expression increased from 6 dpi until 10 dpi (Fig. 2A). In contrast, the expression of the MtNRLK1-related RLK known to be functionally linked to nodulation, *MtSUNN*, was not enhanced during nodulation (Fig. 2B). Hence, *MtNRLK1* and *MtSUNN* expression is differently regulated during nodulation.

### *MtNRLK1* spatial expression pattern is divergent from *MtSUNN* in nodules

**Figure 3:**
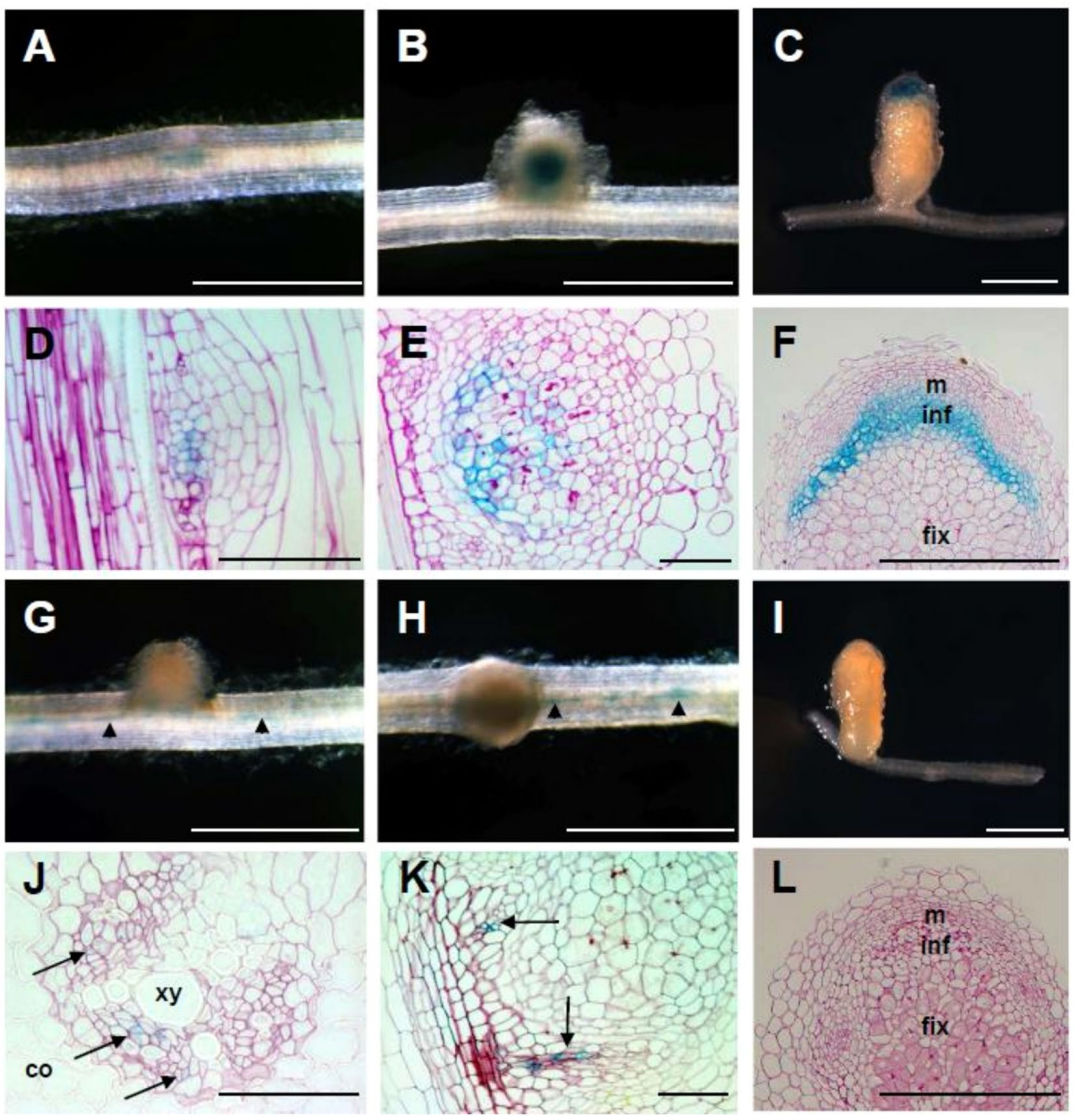
Spatial expression of *MtNRLK1* and *MtSUNN* during nodulation. (**A**–**F**) *Promoter:GUS* activity of *MtNRLK1* in nodule primordia (**a**,**d**, 4 days post-*Rhizobium* inoculation [dpi]), round-shaped nodules (**B**,**E**, 12 dpi) and nitrogen-fixing nodules (**C**,**F**, 24 dpi). (**D**–**F**) are bright-field pictures of longitudinal sections generated through nodules shown in (**A**–**C**), respectively. In nodule primordia, a weak GUS expression is observed at the base of the primordium (**D**). In round-shaped nodules, a blue staining is observed in the central infection zone (**E**). In elongated nodules, expression is restricted to the apical zone (**C**), with the highest expression in the proximal part of the meristem and in the infection zone (**F**). (**G**–**l**), *Promoter:GUS* activity of *MtSUNN* in the Rhizobium-inoculated root stele (**J**), round-shaped nodules (**G**,**H**,**K**, 12 dpi) and elongated nodules (**I**,**L**, 24 dpi). (**J**) is a bright-field picture of a section through a root vascular bundle; (**K**,**L**) are bright-field pictures of longitudinal sections through nodules shown in (**h**) and (**i**), respectively. A blue staining was observed in the phloem of the root vascular bundles (**G**,**H**, arrowheads; **J**, arrows) and at the base of the nodule vascular tissue (**K**, arrows). No expression was detected in the nodule apical meristem, infection and nitrogen fixation zones of round-shaped or elongated nodules (**I**,**L**). m, meristem; inf, infection zone; fix, nitrogen fixation zone, xy, xylem, co, cortex. Bars = 2 mm (**A**–**C**,**G**–**I**), 0.1 mm (**J**), 0.4 mm (**D**,**E**,**K**) and 1 mm (**F**,**L**).

Spatial expression patterns of *MtNRLK1* in uninoculated roots and developing nodules were investigated by *promoter:GUS* analyses and *in situ* hybridizations. A 2-kb region upstream of *MtNRLK1* was fused to the *uidA* gene to monitor transcriptional regulations using a GUS assay. In uninoculated roots, *MtNRLK1* showed a very low expression in the stele, an expression domain similar to the one of *MtSUNN* as previously reported^37^ (Fig. S3). At early stages after rhizobial inoculation, *pMtNRLK1:GUS* activity was observed within the incipient nodule primordia (Fig. 3A,D) but not in infected root hairs, in agreement with Breakspear *et al*.^38^ and Jardinaud *et al*.^39^ transcriptomic datasets. Root sections revealed an expression at the base of the nodule primordium (Fig. 3B,E). In elongated nodules, a *pMtNRLK1:GUS* activity was detected in the apex, with a higher expression in the proximal part of the meristem and in the differentiation/rhizobial infection zone (Fig. 3C,F). *In situ* hybridizations were alternatively used to detect *MtNRLK1* transcript accumulation in differentiated nodules, revealing an expression pattern similar to the one obtained with the *promoter:GUS* fusion (Fig. S4). The *MtNRLK1* pattern is therefore divergent from the *MtSUNN* pattern initially reported in Schnabel *et al*.^37^ and that is fully consistent with our observations linking *MtSUNN* expression to root (Fig. 3J) and nodule vascular tissues (Fig. 3K), but not to central or apical tissues of round-shaped young nodules (Fig. 3G,H,J,K) or of elongated nodules (Fig. 3I,L). Importantly, the divergence between these two expression patterns is independently demonstrated by “digital *in situ*” data generated from RNAseq transcriptomes of laser dissected nodule central tissues^36^ (Fig. S5). In conclusion, *MtNRLK1* and *MtSUNN* have a divergent spatial expression pattern in symbiotic nodules.

### Alterations of *MtNRLK1* expression do not induce any major nodulation phenotype

**Figure 4:**
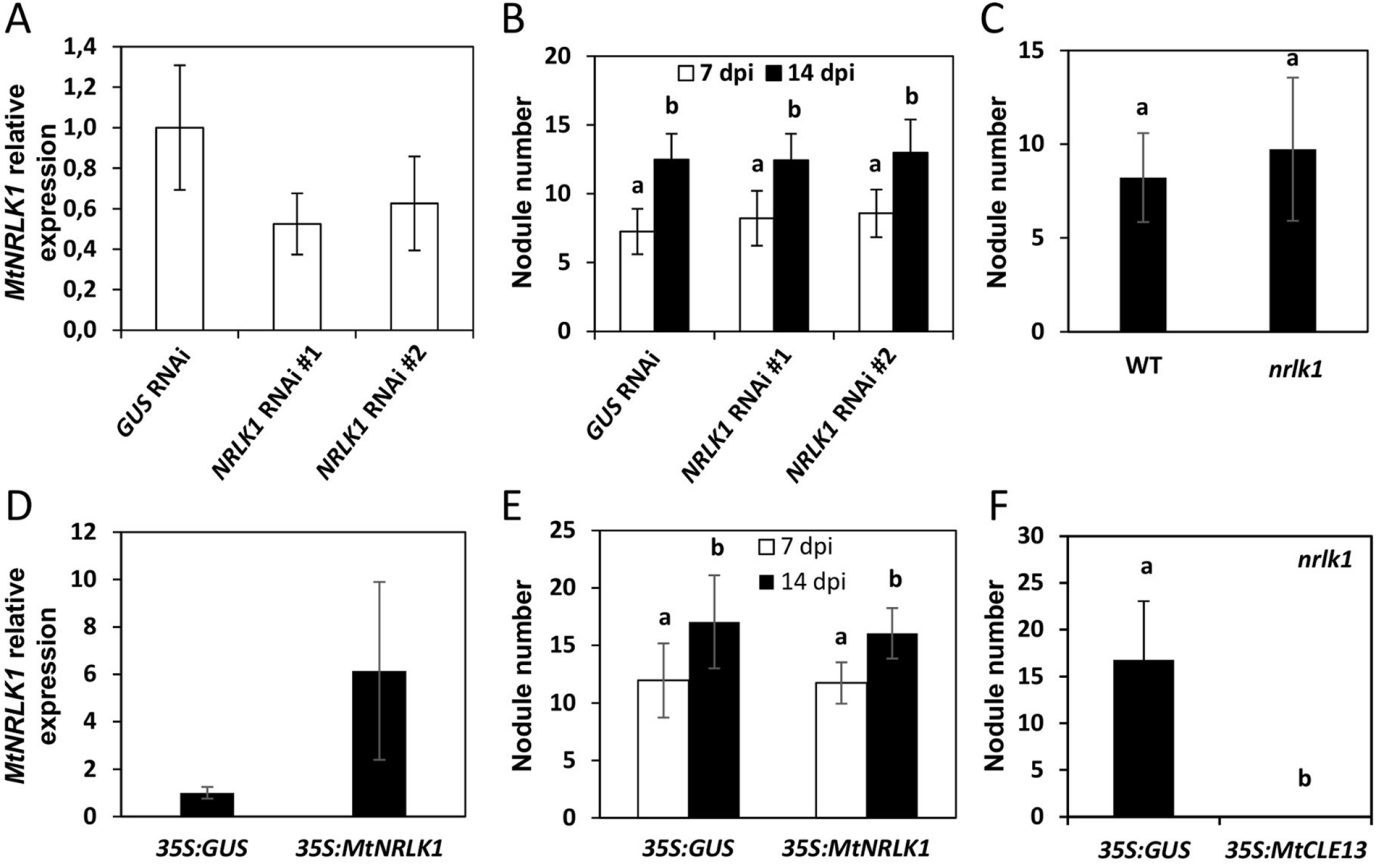
Nodulation phenotype of *MtNRLK1* RNAi or overexpressing roots and of an insertional mutant plant. (**A**,**B**) Nodulation phenotype of roots expressing a RNAi construct to silence *MtNRLK1*. (**A**) Analysis of *MtNRLK1* expression by real-time RT-PCR in roots expressing a *GUS* RNAi (control) or a *MtNRLK1* RNAi construct (#1 or #2). Error bars represent Standard Deviation (SD; n > 4 plants/construct). (**B**) Nodule number in composite plants expressing a *GUS* RNAi or a *MtNRLK1* RNAi construct at 7 and 14 days post-inoculation (dpi). Two independent biological experiments are shown and error bars represent confidence intervals (α = 0.05; n > 30). Letters indicate statistically significant differences (Kruskal-Wallis test, α < 0.05). (**C**) Nodule number in *nrlk1* mutant plants was compared to the Wild-Type (WT) 14 days post-inoculation (dpi). Two biological replicates are shown and error bars represent confidence intervals (α = 0.05) (n > 20). Letters indicate significant differences (Mann & Whitney test, α < 0.05). (**D**,**E**) Nodulation phenotype of roots overexpressing *MtNRLK1*. (**D**) Analysis of *MtNRLK1* expression by real-time RT-PCR in roots overexpressing *GUS* (*35S:GUS*) or *MtNRLK1* (*35S:MtNRLK1*). Error bars represent SD (n > 4). (**E**) Nodule number in *35S:GUS* or *35S:MtNRLK1* composite plants at 7 and 14 days post-inoculation (dpi). Error bars represent confidence intervals (α = 0.05; n > 23). Letters indicate statistically significant differences (Kruskal-Wallis test, α < 0.05). (**F**) Nodulation phenotype of *nrlk1* roots overexpressing GUS or *MtCLE13* at 14 dpi. Two independent experiments are shown and error bars represent confidence intervals (α = 0.05; n > 11). Letters indicate statistically significant differences (Mann & Whitney test, α < 0.05).

In order to investigate the potential function of MtNRLK1 during nodulation, we first attempted to silence the *MtNRLK1* expression in roots by RNA interference (RNAi) using *Agrobacterium rhizogenes* transformation. Two independent RNAi constructs were designed (Fig. S6A). In both cases, roots expressing the RNAi constructs showed a *MtNRLK1* expression level that was about half of the level detected in roots expressing the *GUS* RNAi control (Fig. 4A). No significant difference in nodule number could be however revealed when compared to *GUS* RNAi roots (Fig. 4B).

As RNAi constructs led to a moderate silencing efficiency and only affected the root system, we then identified a *Tnt1* (*Transposable element of Nicotiana tabacum cell type1*) insertional mutant affecting the MtNRLK1 genomic locus. The NF2218 line contains a *Tnt1* insertion located in an exon corresponding to the region encoding the kinase domain of MtNRLK1 (Fig. S6A–C), and therefore predicted to abolish the LRR-RLK activity. No significant difference in the number of nodules formed on *nrlk1* mutant plants was however observed compared to Wild-Type (WT) plants (Fig. 4C). Moreover, no difference in nodule size, organization and nitrogen fixation capacity could be observed between *nrlk1* mutants and WT controls (Fig. S7).

As MtNRLK1 loss of function experiments did not allow to reveal any function for this LRR-RLK in symbiotic nodulation despite its potentially relevant expression pattern, we additionally ectopically expressed *MtNRLK1* in roots. When compared to roots overexpressing the *GUS* control, an about 6 fold higher expression level of *MtNRLK1* was detected in average in *MtNRLK1* overexpressing roots (*MtNRLK1* OE) (Fig. 4D). The number of nodules formed on control roots *versus MtNRLK1* OE roots was however not significantly different (Fig. 4E). Again, no qualitative difference in nodule size or shape could be identified.

Finally, as *MtNRLK1* and *MtSUNN* expression patterns overlap in root vascular tissues, we investigated whether MtNRLK1 could, like MtSUNN, be involved in the systemic AON regulation depending on the CLE12/13 peptides^16^. As previously observed in WT roots, a strong decrease in nodule number was detected in *nrlk1* transgenic roots expressing a *35S:MtCLE13* construct compared to the *35S:GUS* (control) (Fig. 4F). This suggests that MtNRLK1 does not interfere with the inhibition of nodulation provoked by *MtCLE13* overexpression.

## Discussion

The aim of this study was to identify and characterize subclass XI LRR-RLKs closely related to SUNN showing an enhanced expression during *M. truncatula* nodulation. Amongst the 10 closest SUNN proteins, only MtNRLK1 showed a symbiosis-related expression pattern. MtNRLK1 is most closely related to the *A. thaliana* AtBAM3 receptor while MtSUNN is the closest *M. truncatula* homolog of AtCLV1^21^. As both AtBAM3 and AtCLV1 function in SAM homeostasis by regulating stem cell identity^40–42^, we have made the hypothesis that *MtNRLK1* might be involved in controlling the balance between cell proliferation and differentiation within the nodule potentially in relation with MtCLE12 and MtCLE13 peptides previously shown to be expressed in differentiated nodules^16^.

*MtNRLK1* transcripts were detected at the base of nodule primordia, which accordingly to the nodule fate map proposed by Xiao *et al*.^13^ may correspond to cells derived from pericycle and inner cortex root layers, whereas *MtCLE12* and *MtCLE13* are expressed in whole primordia^16^. In addition, no *pMtNRLK1:GUS* activity was observed in infected root hairs, consistently with recent RNAseq analyses focused on the root epidermis or root hairs that revealed no expression of either *MtNRLK1* or *MtSUNN*^38,39^. In differentiated nodules, *MtNRLK1* is mostly expressed in the differentiation/rhizobial infection zone, in agreement with laser dissection transcriptomic datasets of Roux *et al*.^36^, which corresponds to cells derived from the nodule central meristem. This expression domain therefore partially overlaps with the expression pattern of *MtCLE12* and *MtCLE13* GUS transcriptional fusions reported by Mortier *et al*.^16^ in the nodule meristem and in the distal infection/differentiation zone. As in Arabidopsis CLE peptides are known to act as short distance signals that are perceived by neighboring cells^10,43–46^, the *MtCLE12/MtCLE13* and *MtNRLK1* expression domains could be consistent with a short-distance function of MtCLE12 and MtCLE13 peptides in the nodule apex.

No significant nodulation phenotype could be however identified in *MtNRLK1* RNAi, mutant or overexpression lines. This suggests that *MtNRLK1* may act redundantly with other RLKs still to be discovered. In Arabidopsis, *bam3* single mutants also have a WT phenotype suggestive of a functional redundancy^9^. MtSUNN is most likely not a candidate RLK functionally redundant with MtNRLK1 in the nodule apex as no *SUNN* transcripts were detected in the nodule meristem or differentiation/rhizobial infection zones. In Arabidopsis, the expression pattern of AtBAM3 and AtCLV1 is also divergent, even though both receptors were associated to AtCLV3 peptide signaling in the SAM^9^. As MtNRLK1 is closely related to the Medtr3g449390 protein, a functional redundancy may exist between these two receptors despite the expression of this gene is not nodule-enhanced and even lower in nodules than in roots. As the *MtSUNN* expression pattern is redundant with *MtNRLK1* in vascular tissues of the root stele, accordingly to the expression profile reported in Schnabel *et al*.^37^, and for *HAR1* in *L. japonicus* and *NARK* in *Glycine max*^47^, a functionally redundant unknown function of these genes may exist locally in roots. Finally, no functional data currently supports a role of *MtNRLK1* in relation to the *SUNN*-dependent AON systemic pathway inhibiting nodulation depending on CLE12/CLE13 peptides^24^, as a *35S:CLE13* overexpression strongly reduced nodule numbers in *nrlk1* mutants similarly as in WT plants.

Altogether, whereas *MtNRLK1* expression is nodule-enhanced and tightly associated to the nodule apex containing the meristem and the differentiation zone, we could not observe any related symbiotic phenotype when deregulating up or down *MtNRLK1* expression. Future studies, including the functional analysis of additional LRR-RLKs expressed in nodules and the production of double mutants, notably between MtSUNN and MtNRLK1 as well as between Medtr3g449390 and MtNRLK1, may give more insights about possible local and/or systemic functions of MtNRLK1 in symbiotic nodulation.

## Materials and Methods

### Biological material

*Medicago truncatula* Gaertn. cv. Jemalong A17 seedlings were grown and inoculated as previously described^48^. A *nrlk1* mutant (NF2218) was identified from the *Tnt1* insertional mutant collection generated at the Noble Foundation (USA,^49^; http://bioinfo4.noble.org/mutant/). Genotyping of the homozygous mutants was achieved using the LTR4 primer TACCGTATCTCGGTGCTACA and the NF2218-R1 AGCAAGTCCAAAATCAGCAA primer to amplify the *Tnt1* insertion, and the NF2218-F1 primer ATGGTGGTGAGAAACCTGGA and the NF2218-R2 primer TTTCCCATGCAACACTTCAC to amplify the *NRLK1* locus. The *Sinorhizobium meliloti* strain *Sm1021*^50,51^ was grown at 28 °C in a Yeast Extract Broth medium (YEB^52^, supplemented with 10 mg L^−1^ tetracycline.

For *promoter:GUS* clonings, a 2-kb region upstream of *MtNRLK1* (Medtr5g090100) and *MtSUNN* (Medtr4g070970, *M. truncatula* genome v4.0, JCVI http://jcvi.org/medicago/) was isolated from genomic DNA. The promoters were fused to the *uidA* gene in the pKm43GWRolD vector^53^. Primers used for amplification are listed in Table S1.

For quantitative RT-PCR (qRT-PCR) analyses, *M. truncatula* plants were grown *in vitro* in square Petri dishes on a nitrogen-poor solution ‘i’ with Kalys agar^54^. For the nodulation kinetic, susceptible root zones were harvested before inoculation and at 4, 6, 8 and 10 dpi with *Sm2011* from plants grown in pouches and watered with nitrogen-poor solution ‘i’ medium. Infection threads were visible from 4 dpi on, nodule primordia at 6 dpi, and young nodules at 8 and 10 dpi. Tissue was collected by visualizing the green fluorescent bacteria under a stereomicroscope MZFLII (Leica Microsystems, Wetzlar, Germany) equipped with a blue-light source and a Leica GFP Plus filter set (λ_ex_ = 480/40; λ_em_ = 510 nm LP barrier filter). For the *nrlk1* phenotype analysis, plants were grown in a mixture of perlite/sand (3/1) and watered with the nitrogen-poor solution ‘i’. Inoculation with *Sm1021* was performed 7 days post-germination and nodules were scored 15 dpi. To evaluate the nodule nitrogen-fixation capacity and histology, a more efficient nitrogen-fixing strain was used, *S. medicae* WSM419. Sections were obtained using a vibratome (Leica VTS1200) on agarose 1% embedded nodules (3 weeks post-inoculation) and imaged with a Fluo AZ100 macroscope (Nikon). Acetylene reduction assays were used to evaluate the nitrogen fixation capacity, as previously described^31^.

### Protein similarity tree analysis

To identify MtSUNN-related LRR-RLKs, the full amino acid sequence of MtSUNN was blasted on the *Medicago truncatula* Proteome v4 database using the BLASTP algorithm (http://www.jcvi.org/medicago/). In addition, the 10 proteins most closely related to MtNRLK1 in *A. thaliana* were retrieved from the TAIR10 protein database (https://www.arabidopsis.org/) by using the BLASTP algorithm. The MtCRA2 LRR-RLK, previously functionally characterized in *M. truncatula* regarding symbiotic nodulation, was also included. To generate a similarity tree using the SeaView version 4 program^55^, complete amino acid sequences of MtSUNN and MtNRLK1 as well as the *A. thaliana* and *M. truncatula* closest related sequences previously identified were used to generate a multiple alignment using MUSCLE^56^. Poorly aligned positions and divergent regions were eliminated with the G-blocks software^57^. A maximum-likelihood phylogenetic tree was obtained using the PhyML program^58^ as well as an aLRT (approximate Likelihood-Ratio Test) test for branch support.

### RNA extraction, cDNA synthesis, and qRT-PCR analysis

Total RNA was isolated with the RNeasy Plant mini kit (Qiagen, Hilden, Germany) according to the manufacturer’s instructions. After a DNAse treatment, samples were purified through NH_4_ acetate (5 M) precipitation, quality controlled, and quantified with a Nanodrop spectrophotometer (Isogen, Hackensack, NJ). RNA (2 μg) was used for cDNA synthesis with the Superscript Reverse Transcriptase Kit (Invitrogen, Carlsbad, CA). The samples were diluted 50 times and the qRT-PCR experiments were performed on a LightCycler 480 (Roche Diagnostics, Brussels, Belgium) using the LightCycler480 SYBR Green I Master Kit according to manufacturer’s instructions (Roche). All reactions were performed in triplicate and averaged. The relative expression was normalized against the reference genes Histone 3-Like (H3L, Medtr4g097170) or a 40S Ribosomal S8 Protein (TC100533, *M. truncatula* Gene Index version 8). Primers used are indicated in Table S1.

### Agrobacterium rhizogenes-mediated root transformation

The protocol was adapted from Boisson-Dernier *et al*.^59^. Approximately 48 h after germination, radicles of WT or *nrlk1* seedlings were sectioned 5 mm above the root tip with a sterile scalpel. Sectioned seedlings were infected by coating the freshly cut surface with an *A. rhizogenes* Arqua1 strain containing the vector of interest (CLE13 OE^16^). The *A. rhizogenes* strain was grown at 28 °C for two days on a solid agar-YEB medium supplemented with spectinomycin (100 µg/ml)^60^. Infected seedlings were grown on a Fahraeus medium supplemented with 1 mM NH_4_NO_3_ and kanamycin (25 mg/L^61^;) in square Petri dishes for one week at 20 °C, 16-h photoperiod (150 μE m^−2^ s^−1^) then for one more week at 24 °C under identical light conditions. Subsequently, composite plants selected on kanamycin were transferred on a brown growth paper (Mega international, http://www.mega-international.com/) on a Fahraeus medium without nitrogen, grown at 24 °C under identical light conditions for five days, and then inoculated with *S. meliloti Sm1021* strain at OD_600nm_ = 0.05.

### Histochemical localization of GUS activity

GUS activity in transformed roots and nodules was analyzed using 5-bromo-4-chloro-3-indolyl-β-d-glucuronic acid as a substrate. Roots and nodules were vacuum infiltrated during 20 min and subsequently incubated in a GUS buffer at 37 °C. Incubation lasted 6 h and 10 h for *pMtNRLK1-GUS* and *pMtSUNN-GUS*, respectively. After staining, roots and root nodules were fixed, dehydrated, embedded with Technovit 7100 (Heraeus Kulzer, Wehrheim, Germany), according to the manufacturer’s instructions, and sectioned with a microtome (Reichert-Jung, Nussloch, Germany). Three μm thick sections were mounted on coated slides (Sigma-Aldrich, St. Louis, MO). For tissue counter staining, sections were treated with a 0.05% (w/v) ruthenium red solution (Sigma-Aldrich, St. Louis, MO), washed in distilled water, and dried. Sections were mounted with Depex (BDH Chemicals, Poole, England), and pictures were taken with a Diaplan microscope equipped with bright- and dark-field optics (Leitz, Wetzlar, Germany).

### *In situ* hybridization

Ten μm sections obtained with a microtome (Reichert-Jung, Nussloch, Germany) on paraffin-embedded nodules were hybridized as previously described^62^. A ^35^S-labeled antisense probe, corresponding to a region spanning the open reading frame and the 3′ UTR of *MtNRLK1*, was produced using the T7 RNA polymerase (Invitrogen) on a PCR-amplified probe (primers listed in Table S1).

### Statistical analyses

Non-parametric tests were used to assess significant differences: a Mann and Whitney test when two conditions are compared and a Kruskal-Wallis test when more than two conditions are considered.

## Electronic supplementary material

Supplementary Information
