# Supplementary material for: MtNRLK1, a CLAVATA1-like leucine-rich repeat receptor-like kinase upregulated during nodulation in Medicago truncatula
Source: Sci Rep. 2018 Feb 1;8:2046. doi: 10.1038/s41598-018-20359-4 (PMC5794917; doi:10.1038/s41598-018-20359-4)
Supplement: Supplementary file 1 — Supplementary Information [file 41598_2018_20359_MOESM1_ESM.pdf]

MtNRLK1*, a CLAVATA1-like leucine-rich repeat receptor-like kinase upregulated during nodulation in *Medicago truncatula

Carole Laffont, Carolien De Cuyper, Justine Fromentin, Virginie Mortier, Annick De Keyser, Christa Verplancke, Marcelle Holsters, Sofie Goormachtig, Florian Frugier

```
                1
MtNRLK1         -------MDT PSFTFVLYTL FLTLSVSVSS SSSL------ P-MSLKTQAS ILVSLKQDFE ----SKTS-L KSWN--ISNY MS-----LCT TWYGIQCD--
Medtr3g44930    ---------- ---MATPLIF YLLLVSFTSF SFVS-----S LQLSLKNQAS ILVSMKQDFE ---PSSNTSL SSWN--MSNY MS-----LC- TWYGIQCDHT
Medtr2g005810   ---------- ----MIPLTL TVFTLFSVLF SSVS------ A-SSLLSDFH ALVTLRQGFQ ---FPNPV-I NTWN--TSNF SS-----VC- SWVGIQCH--
Medtr5g014700   ---------- -----MRFLL LLFFLFHFHY HHVL------ SASAPISEYR ALLSFRQSIT ---DSTPPSL SSWN--TNTT -------HC- TWFGVTCN--
Medtr4g097880   ---------- ----MLPFLP LLLFLLSFNL NQVL------ S-TPHISEYH SLLSFKSSIT --NDPQNI-L TSWN--PKT- -P-----YC- SWYGIKCS--
SUNN            ---------- ---------- ---------- ---------- ---------- ----MKGE-- ---KAKDDAL KDWK-FSTSA SA-----HC- SFSGVKCD--
CRA2            ---------- --MNHHQPFF FFGLIVIISM TNMS------ -HVISTNQSE FFSLMKESLS ---GNYPF-- -DWG-VSKVD KP-----IC- DFTGITCD--
Medtr1g069470   ---------- ------MQLK TQSFIFFFFC CLCC------ AGAAADNEAF ALLSIKAGLI ---DPLNS-L HDWK-DGGAA QA-----HC- NWTGVQCN--
Medtr7g098610   ----MRMKNN MMQMKTQIFI FFCYIVIFCF SNSF------ -SAASNDEVS ALLSLKEGLV ---DPLNT-L QDWK--LDAA -------HC- NWTGIECN--
Medtr1g080440   ---------- ---MQTHLFL FYCYIIVSLI FTER------ AQSATNDELS TLLSIKSSLI ---DSMNH-L KDWQ-PPSNA TRWQSRLHC- NWTGIGCN--
Medtr4g03675    ---------- -----MKPFL FFLITFLFFS QTQLLPLVSS ATTTIPFQLI SLLSIKSSLI ---DPLNH-L NDWKNNPSDS NNQQDPIWC- SWTGINCH--
BAM3            ---------- MADKIFTFFL ILSSISPLLC SSLI----SP LNLSLIRQAN VLISLKQSFD ---SYDPS-L DSWN--IPNF NS-----LC- SWTGVSCD--
BAM1            ---------- -------MKL FLLLLFLLHI SHTF------ TASRPISEFR ALLSLKTSLT GAGDDKNSPL SSWK--VSTS -------FC- TWIGVTCD--
BAM2            ---------- -----MKLLL LLLLLLLLHI SHSF------ TVAKPITELH ALLSLKSSFT --IDEHSPLL TSWN--LSTT -------FC- SWTGVTCD--
CLV1            ---------M AMRLLKTHLL FLHLYLFFSP CFAY------ ------TDME VLLNLKSSMI ---GPKGHGL HDWI-HSSSP DA-----HC- SFSGVSCD--
PXY             -----MKKKN ISPSLVLHPL LLLLLPFFAF NSLA------ --LKFSPQLL SLLSLKTSLS ---GPPSA-F QDWK-VPVNG QN--DAVWC- SWSGVVCD--
AT4G28650       ---------- --MKMKIIVL FLYYCYIGST SSVL------ ASIDNVNELS VLLSVKSTLV ---DPLNF-L KDWK--LSDT SD-----HC- NWTGVRCN--
AT1G08590       ---------- --MAIPRLFF LFYYIGFALF PFVS------ SETFQNSEQE ILLAFKSDLF ---DPSNN-L QDWK-RPENA TTFSELVHC- HWTGVHCD--
AT3G24240       ----MSLHSL IFFSSSSSSL LFSFFFIFIF CFSL------ SDAEQNPEAS ILYSWLHSSS --PTPSSLSL FNWN--SIDN TP------CN NWTFITCS--
HSL1            ---------- ------MYLL FLFLLFPTVF SLNQ------ -------DGF ILQQVKLSLD ---DPDSY-L SSWN--SNDA SP------C- RWSGVSCA--
AT5G48940       MSLQMPIPRK KALTVSHFSI TLSLFLAFFI SSTS------ ---ASTNEVS ALISWLHSSN ---SPPPSVF SGWN--PSDS DP------C- QWPYITCS-S
AT2G25790       -------MST SHHHHHPPYL ITTLFFLFLN FSCL------ ----HANELE LLLSFKSSIQ ---DPLKH-L SSWS--YSST ND-----VC- LWSGVVCN--

               101
MtNRLK1         TNNSSVVSLD ISN-LNVSGT -FSSSITKLS ---------- ---------- -------NLR FLNISNN-MF NGNL-SWKFS H-LKELEVLD AYNNEFNCSL
Medtr3g44930    ITNMSIVSLD ISN-LNISGS -FSPQITKLY -NLVNVSIQG NSFYGEFPTE IHKLQ--RLK CLNISNN-MF SGNL-SWEFN K-LKELEVLD IYNNGFNGSL
Medtr2g005810   --QGRVVSLD LTD-LNLFGS -VSPSISSLD -RLSHLSLAG NNFTGTI--H ITNLT--NLQ FLNISNN-QF SGHM-DWNYS T-MENLQVVD VYNNNFTSLL
Medtr5g014700   -TRRHVTAVN LTG-LDLSGT -LSDELSHLP -FLTNLSLAD NKFSGQIPPS LSAVT--NLR LLNLSNN-VF NGTF-PSELS L-LKNLEVLD LYNNNMTGTL
Medtr4g097880   -QHRHVISLN LTS-LSLTGT -LS--LSNLP -FLTNLSLAD NKFSGPIPSS LSSLS--SLR FLNLSNN-IF NGTL-PQELS N-LFNLQVLD LYNNNMTGSL
SUNN            -EDQRVIALN VTQ-VPLFGH -LSKEIGELN -MLESLTITM DNLTGELPTE LSKLT--SLR ILNISHN-LF SGNF-PGNIT FGMKKLEALD AYDNNFEGPL
CRA2            -NKGDIISLD FSGWSSLSGN -FPSNICSYL PNLRVLNLGN TKFKFP-TNS IINCS--HLE LLNMNKM-HL SGTL-P-DFS S-LKYLRVLD LSYNSFTGDF
Medtr1g069470   -SAGAVEKLN LSH-MNLSGS -VSNEIQSLK -SLTFLNLCC NGFESSLSKH ITNLT--SLK SLDVSQN-FF TGGF-PLGLG K-ASELLTLN ASSNNFSGFL
Medtr7g098610   -SAGTVENLD LSH-KNLSGI -VSGDIQRLQ -NLTSLNLCC NAFSSPFPKF ISNLT--TLK SLDVSQN-FF IGEF-PLGLG K-ASGLTTLN ASSNEFTGSI
Medtr1g080440   -TKGFVESLE LYN-MNLSGI -VSNHIQSLS -SLSYFNISC NNFASTLPKS LSNLT--SLK SFDVSQN-YF TGTF-PTGFG R-AAELKSIN ASSNEFSGLL
Medtr4g03675    PKTAQITSLN LSN-LNLSGI -ISPKIRYLT -TLTHLNISG NDFNGTFQTA IFQLN--ELR TLDISHN-SF NSTF-PPGIS K-LRFLRVFN AYSNSFVGPL
BAM3            NLNQSITRLD LSN-LNISGT -ISPEISRLS PSLVFLDISS NSFSGELPKE IYELS--GLE VLNISSN-VF EGELETRGFS Q-MTQLVTLD AYDNSFNGSL
BAM1            VSRRHVTSLD LSG-LNLSGT -LSPDVSHLR -LLQNLSLAE NLISGPIPPE ISSLS--GLR HLNLSNN-VF NGSF-PDEIS SGLVNLRVLD VYNNNLTGDL
BAM2            VSLRHVTSLD LSG-LNLSGT -LSSDVAHLP -LLQNLSLAA NQISGPIPPQ ISNLY--ELR HLNLSNN-VF NGSF-PDELS SGLVNLRVLD LYNNNLTGDL
CLV1            -DDARVISLN VSF-TPLFGT -ISPEIGMLT -HLVNLTLAA NNFTGELPLE MKSLT--SLK VLNISNNGNL TGTF-PGEIL KAMVDLEVLD TYNNNFNGKL
PXY             NVTAQVISLD LSH-RNLSGR -IPIQIRYLS -SLLYLNLSG NSLEGSFPTS IFDLT--KLT TLDISRN-SF DSSF-PPGIS K-LKFLKVFN AFSNNFEGLL
AT4G28650       -SNGNVEKLD LAG-MNLTGK -ISDSISQLS -SLVSFNISC NGFESLLPKS IP-----PLK SIDISQN-SF SGSL-FLFSN E-SLGLVHLN ASGNNLSGNL
AT1G08590       -ANGYVAKLL LSN-MNLSGN -VSDQIQSFP -SLQALDLSN NAFESSLPKS LSNLT--SLK VIDVSVN-SF FGTF-PYGLG M-ATGLTHVN ASSNNFSGFL
AT3G24240       -SQGFITDID IES-VPLQLS -LPKNLPAFR -SLQKLTISG ANLTGTLPES LGDCL--GLK VLDLSSN-GL VGDI-PWSLS K-LRNLETLI LNSNQLTGKI
HSL1            GDFSSVTSVD LSS-ANLAGP -FPSVICRLS -NLAHLSLYN NSINSTLPLN IAACK--SLQ TLDLSQN-LL TGEL-PQTLA D-IPTLVHLD LTGNNFSGDI
AT5G48940       SDNKLVTEIN VVS-VQLALP -FPPNISSFT -SLQKLVISN TNLTGAISSE IGDCS--ELI VIDLSSN-SL VGEI-PSSLG K-LKNLQELC LNSNGLTGKI
AT2G25790       -NISRVVSLD LSG-KNMSGQ ILTAATFRLP -FLQTINLSN NNLSGPIPHD IFTTSSPSLR YLNLSNN-NF SGSI-PRGF- --LPNLYTLD LSNNMFTGEI
```

```
                 401
MtNRLK1          PLGVTELP-K LKYLNFGGNF FYGEIPSKYG NMLQLNYLSL AGNDLR-GFI PFELGNLTNL THLLLG---- ---------- ---------Y YNEFDGEIPP
Medtr3g44930     PRGVTQVS-S LKHLNFGGNY FSGKIPTSYG EMKQLNFLSL AGNDLS-GFL PSELGNLTSL ENLYLG---- ---------- ---------Y FNQFDGGVPK
Medtr2g005810    PLGILSLKNK LKHLDLGGNF FFGEIPKSYG KLVSLEYLSL AGNDIS-GKI PGELGNLSNL REIYLG---- ---------- ---------Y YNTYEGGIPM
Medtr5g014700    PLAVTELP-N LRHLHLGGNY LTGQIPPEYG SWQHLQYLAV SGNELD-GTI PPEIGNLTSL RELYIG---- ---------- ---------Y FNEYTGGIPP
Medtr4g097880    PVSVTHLS-F LRHLHLGGNF FTGKIPPEYG SWTHLEYLAV SGNELS-GHI PPEIGNITSL KELYIG---- ---------- ---------Y YNTYDGGIPP
SUNN             PEEIVSLM-K LKYLSFAGNF FSGTIPESYS EFQKLEILRL NYNSLT-GKI PKSLSKLKML KELQLG---- ---------- ---------Y ENAYSGGIPP
CRA2             PMSVFNLT-N LEILNFNEN- ---------- ---------- --SKLNLWEL PKSFVRLRSL KSMILS---- ---------- ---------T -CMLHGQIPP
Medtr1g069470    PEDLGNIS-S LETLDLRGSF FEGSIPKSIS NLSNLKYLGL SGNNLT-GKI PAEIGKLSSL EYMIIG---- ---------- ---------Y -NEFEGGIPK
Medtr7g098610    PLDIGNAT-S LEMLDLRGSF FEGSIPKSFS NLHKLKFLGL SGNNLT-GKI PGELGNLSSL EYMILG---- ---------- ---------Y -NEFEGEIPA
Medtr1g080440    PEDIENAT-L LESFDFRGNY FASPIPKSFK NLQKLKFLGL SGNNFT-GKI PEYLGELSSL ETLIMG---- ---------- ---------Y -NAFEGEIPA
Medtr4g03675     PEEFIRLP-F LEHLNLGGSY FSGKIPQSYG TFKRLKFLYL AGNALE-GSL PPQLGLLSEL QRLEIG---- ---------- ---------Y -NSYSGAIPV
BAM3             PLSLTTLT-R LEHLDLGGNY FDGEIPRSYG SFLSLKFLSL SGNDLR-GRI PNELANITTL VQLYLG---- ---------- ---------Y YNDYRGGIPA
BAM1             PVSVTNLT-Q LRHLHLGGNY FAGKIPPSYG SWPVIEYLAV SGNELV-GKI PPEIGNLTTL RELYIG---- ---------- ---------Y YNAFEDGLPP
BAM2             PVSLTNLT-Q LRHLHLGGNY FSGKIPATYG TWPVLEYLAV SGNELT-GKI PPEIGNLTTL RELYIG---- ---------- ---------Y YNAFENGLPP
CLV1             PPEMSELK-K LKYLSFGGNF FSGEIPESYG DIQSLEYLGL NGAGLS-GKS PAFLSRLKNL REMYIG---- ---------- ---------Y YNSYTGGVPP
PXY              PSDVSRLR-F LEELNFGGSY FEGEIPAAYG GLQRLKFIHL AGNVLG-GKL PPRLGLLTEL QHMEIG---- ---------- ---------Y -NHFNGNIPS
AT4G28650        TEDLGNLV-S LEVLDLRGNF FQGSLPSSFK NLQKLRFLGL SGNNLT-GEL PSVLGQLPSL ETAILG---- ---------- ---------Y -NEFKGPIPP
AT1G08590        PEDLGNAT-T LEVLDFRGGY FEGSVPSSFK NLKNLKFLGL SGNNFG-GKV PKVIGELSSL ETIILG---- ---------- ---------Y -NGFMGEIPE
AT3G24240        PPDISKCS-K LKSLILFDNL LTGSIPTELG KLSGLEVIRI GGNKEISGQI PSEIGDCSNL TVLGLAETSV SGNLPSSLGK LKKLETLSIY TTMISGEIPS
HSL1             PASFGKFE-N LEVLSLVYNL LDGTIPPFLG NISTLKMLNL SYNPFSPSRI PPEFGNLTNL EVMWLT---- ---------- ---------E -CHLVGQIPD
AT5G48940        PPELGDCV-S LKNLEIFDNY LSENLPLELG KISTLESIRA GGNSELSGKI PEEIGNCRNL KVLGLAATKI SGSLPVSLGQ LSKLQSLSVY STMLSGEIPK
AT2G25790        YNDIGVFS-N LRVLDLGGNV LTGHVPGYLG NLSRLEFLTL ASNQLT-GGV PVELGKMKNL KWIYLG---- ---------- ---------Y -NNLSGEIPY

                 301
MtNRLK1          HFGNLVNLVH LDLANCGLKG SIPHELGKLY KLDTLFLQTN QL-NGSIPPQ LGNLSSLKSL DMSNNELNGN IPNEFSNLRE LTLLNLFINK LYGEIPSFF-
Medtr3g44930     EFGKLINLVH LDLASCFLKG SIPLELGQLN KLDTLFLQKN QL-TGFIPPE LGNLSRLNAL DLSLNNLTGG IPNEFSNLRE LSLLNLFINK FHSEIPDFI-
Medtr2g005810    EFGRLTKLVH MDISSCDLDG SIPRELGNLK ELNTLYLHIN QL-SGSIPKQ LGNLTNLLYL DLSSNALTGE IPIEFINLNR LTLLNLFLNR LHGSIPDYI-
Medtr5g014700    QIGNLTELIR LDAAYCGLSG EIPHEIGKLQ NLDTLFLQVN AL-SGSLTWE LGNLKSLKSM DLSNNMLTGE IPTSFGELKN LTLLNLFRNK LHGAIPEFI-
Medtr4g097880    EIGNLSEMVR FDAAYCGLTG EVPPELGKLQ KLDTLFLQVN AL-SGSLTSE LGNLKSLKSM DLSNNAFTGE VPVSFAELKN LTLLNLFRNK LHGAIPEFI-
SUNN             ELGSIKSLRY LEISNANLTG EIPPSLGNLE NLDSLFLQMN NL-TGTIPPE LSSMRSLMSL DLSINGLSGE IPETFSKLKN LTLINFFQNK LRGSIPAFI-
CRA2             SISNITTLID LELSGNFLTG QIPKELGLLK NLQQLELYYN YFLVGSIPEE LGNLTELVDL DMSVNKLTGT IPSSVCKLPK LQVLQFYNNS LTGEIPKSI-
Medtr1g069470    EFGNLTKLKY LDLAEGNVGG EIPDELGKLK LLNTVFLYKN SF-EGKIPTN IGNMTSLVLL DLSDNMLSGN IPAEISQLKN LQLLNFMRNK LSGPVPSGL-
Medtr7g098610    EFGNLTSLKY LDLAVANLGG EIPEELGNLK LLDTLFLYNN NL-EGRIPSQ IGNITSLQFL DLSDNNLSGK IPDEMSLLKN LKLLNFMGNQ LSGFVPSGL-
Medtr1g080440    EFGNMTNLQY LDLAVGTLSG RIPPELGKLK NLTTIYLYRN KF-TAKIPPQ LGNIMSLAFL DLSDNQITGE IPEELAKLEN LQLLNLMSNK LTGPVPKKL-
Medtr4g03675     ELTMLSNLKY LDISGANISG QVIPELGNLS MLETLLLFKN HL-HGEIPSS IGKLKSLQAL DLSENELTGS IPSEITMLKE IVDLRLMYNK LKGEIPQEI-
BAM3             DFGRLINLVH LDLANCSLKG SIPAELGNLK NLEVLFLQTN EL-TGSVPRE LGNMTSLKTL DLSNNFLEGE IPLELSGLQK LQLFNLFFNR LHGEIPEFV-
BAM1             EIGNLSELVR FDGANCGLTG EIPPEIGKLQ KLDTLFLQVN VF-SGPLTWE LGTLSSLKSM DLSNNMFTGE IPASFAELKN LTLLNLFRNK LHGEIPEFI-
BAM2             EIGNLSELVR FDAANCGLTG EIPPEIGKLQ KLDTLFLQVN AF-TGTITQE LGLISSLKSM DLSNNMFTGE IPTSFSQLKN LTLLNLFRNK LYGAIPEFI-
CLV1             EFGGLTKLEI LDMASCTLTG EIPTSLSNLK HLHTLFLHIN NL-TGHIPPE LSGLVSLKSL DLSINQLTGE IPQSFINLGN ITLINLFRNN LYGQIPEAI-
PXY              EFALLSNLKY FDVSNCSLSG SLPQELGNLS NLETLFLFQN GF-TGEIPES YSNLKSLKLL DFSSNQLSGS IPSGFSTLKN LTWLSLISNN LSGEVPEGI-
AT4G28650        EFGNINSLKY LDLAIGKLSG EIPSELGKLK SLETLLLYEN NF-TGTIPRE IGSITTLKVL DFSDNALTGE IPMEITKLKN LQLLNLMRNK LSGSIPPAI-
AT1G08590        EFGKLTRLQY LDLAVGNLTG QIPSSLGQLK QLTTVYLYQN RL-TGKLPRE LGGMTSLVFL DLSDNQITGE IPMEVGELKN LQLLNLMRNQ LTGIIPSKI-
AT3G24240        DLGNCSELVD LFLYENSLSG SIPREIGQLT KLEQLFLWQN SL-VGGIPEE IGNCSNLKMI DLSLNLLSGS IPSSIGRLSF LEEFMISDNK FSGSIPTTIS
HSL1             SLGQLSKLVD LDLALNDLVG HIPPSLGGLT NVVQIELYNN SL-TGEIPPE LGNLKSLRLL DASMNQLTGK IPDELCRVP- LESLNLYENN LEGELPASI-
AT5G48940        ELGNCSELIN LFLYDNDLSG TLPKELGKLQ NLEKMLLWQN NL-HGPIPEE IGFMKSLNAI DLSMNYFSGT IPKSFGNLSN LQELMLSSNN ITGSIPSILS
AT2G25790        QIGGLSSLNH LDLVYNNLSG PIPPSLGDLK KLEYMFLYQN KL-SGQIPPS IFSLQNLISL DFSDNSLSGE IPELVAQMQS LEILHLFSNN LTGKIPEGV-
```

```
             201
MtNRLK1      ---------- ---------- ---SELPNLE VLKLWQNNFT GSIPSKLGKN GKLSELDLST NKLTGLVPKS LCLGKRLKIL ILLNNFLFGS LPNEFGQCYT
Medtr3g44930 ---------- ---------- ---SELPKLE VLKLWRNNFT GVIPSKLGQN GRLTEVDLST NKLTGILPKS LCFGKRLKIL ILLNNFLFGS LPNDLGQCYT
Medtr2g005810 ---------- ---------- ---ADFPDLD TLGLWMNNFT GEIPYKLGLN GKLQILDLSS NKLTGIIPPH LCSSSQLKIL ILLNNFLFGP IPQGLGTCYS
Medtr5g014700 ---------- ---------- ---GDMPALE VIQLWENNFT GNIPMSLGTN GKLSLLDISS NKLTGTLPPY LCSGNMLQTL ITLGNFLFGP IPESLGGCES
Medtr4g097880 ---------- ---------- ---GEMPSLE VLQIWENNFT GSIPQSLGKN GKLTLVDVSS NKLTGSLPPF MCFGNKLQTL IALGNFLFGP IPDSLGKCKS
SUNN         ---------- ---------- ---GDLPNLE TLQVWENNFS FVLPQNLGSN GKFIYFDVTK NHLTGLIPPE LCKSKKLKTF IVTDNFFRGP IPNGIGPCKS
CRA2         ---------- ---------- ---ENSKTLR ILSLYDNFLS GHVPAKLGQS SGMVVLDLSE NKLSGPLPEH VCQGGKLLYF LVLDNFFSGV IPESYSNCMF
Medtr1g069470 ---------- ---------- ---GDLPQLE VLELWNNSLS GPLPRDLGKN SPLQWLDVSS NSLSGEIPET LCTKGNLTKL ILFNNAFKGP IPTSLSKCPS
Medtr7g098610 ---------- ---------- ---GNLPQLE VFELWNNSLS GPLPSNLGEN SPLQWLDVSS NSLSGEIPET LCSKGNLTKL ILFNNAFSGP IPSSLSMCSS
Medtr1g080440 ---------- ---------- ---GELKKLQ VLELWKNSLE GSLPMNLGRN SPLQWLDVSS NSLSGEIPPG LCTTGNLTKL ILFNNSFSGP IPSGLSNCSS
Medtr4g03675 ---------- ---------- ---GDLPKLN TFHIFNNSFT GALPPKLGSN GLLQLLDVST NSLQGSIPIN ICKGNNLVKF NIFNNKFTNN LPSSLTNCTS
BAM3         ---------- ---------- ---SELPDLQ ILKLWHNNFT GKIPSKLGSN GNLIEIDLST NKLTGLIPES LCFGRRLKIL ILFNNFLFGP LPEDLGQCEP
BAM1         ---------- ---------- ---GDLPELE VLQLWENNFT GSIPQKLGEN GKLNLVDLSS NKLTGTLPPN MCSGNKLETL ITLGNFLFGS IPDSLGKCES
BAM2         ---------- ---------- ---GEMPELE VLQLWENNFT GSIPQKLGEN GRLVILDLSS NKLTGTLPPN MCSGNRLMTL ITLGNFLFGS IPDSLGKCES
CLV1         ---------- ---------- ---GELPKLE VFEVWENNFT LQLPANLGRN GNLIKLDVSD NHLTGLIPKD LCRGEKLEML ILSNNFFFGP IPEELGKCKS
PXY          ---------- ---------- ---GELPELT TLFLWNNNFT GVLPHKLGSN GKLETMDVSN NSFTGTIPSS LCHGNKLYKL ILFSNMFEGE LPKSLTRCES
AT4G28650    ---------- ---------- ---SSLAQLQ VLELWNNTLS GELPSDLGKN SPLQWLDVSS NSFSGEIPST LCNKGNLTKL ILFNNTFTGQ IPATLSTCQS
AT1G08590    ---------- ---------- ---AELPNLE VLELWQNSLM GSLPVHLGKN SPLKWLDVSS NKLSGDIPSG LCYSRNLTKL ILFNNSFSGQ IPEEIFSCPT
AT3G24240    NCSSLVQLQL DKNQISGLIP SELGTLTKLT LFFAWSNQLE GSIPPGLADC TDLQALDLSR NSLTGTIPSG LFMLRNLTKL LLISNSLSGF IPQEIGNCSS
HSL1         ---------- ---------- ---ALSPNLY EIRIFGNRLT GGLPKDLGLN SPLRWLDVSE NEFSGDLPAD LCAKGELEEL LIIHNSFSGV IPESLADCRS
AT5G48940    NCTKLVQFQI DANQISGLIP PEIGLLKELN IFLGWQNKLE GNIPDELAGC QNLQALDLSQ NYLTGSLPAG LFQLRNLTKL LLISNAISGV IPLEIGNCTS
AT2G25790    ---------- ---------- ---TSLPRLK VLQLWSNRFS GGIPANLGKH NNLTVLDLST NNLTGKLPDT LCDSGHLTKL ILFSNSLDSQ IPPSLGMCQS

             501
MtNRLK1      LQRVRLGQNY LTGSIPKGFL YLPQLSLLEL QNNLLGGFLP QQEITNTNTS KLGEINLSNN RLSGSLPNSI GNFPNLQILL LHGNRFSGEI PSDIGKLKNI
Medtr3g44930 LQRVRIGQNY FTGSIPHGFL YLPNLSLLEL QNNYLSGVIP QQTHKN-KTS KLEQCNLSNN RLSGSLPTSI GNFPNLQTLQ LSGNRFSGQI PSDIGKLKKI
Medtr2g005810 LTRVRLGENY LNGSIPNGFL YLPKLNLAEL KNNYLSGTLS ENGNSSSKPV SLEQLDLSNN ALSGPLPYSL SNFTSLQILL LSGNQFSGPI PPSIGGLNQV
Medtr5g014700 LTRIRMGENF FNGSIPKGLF GLPKLSQVEL QDNYLSGNFP ETHSV---SV NLGQITLSNN QLSGPLPPSI GNFSGVQKLL LDGNMFEGKI PSQIGRLQQL
Medtr4g097880 LNRIRMGENF LNGSIPKGLF GLPELTQVEL QDNLLSGNFP QPVSM---SI NLGQVTLSNN KLSGPLPPSI GNFTSVQKLI LDGNQFSGKI PAEIGKLHQL
SUNN         LEKIRVANNY LDGPVPPGIF QLPSVQIIEL GNNRFNGQLP TEISG----N SLGNLALSNN LFTGRIPASM KNLRSLQTLL LDANQFLGEI PAEVFALPVL
CRA2         LLRFRVSNNR LEGSVPKGLL SLSHVSIIDL SSNNLTGPI- ---------- ---------- ------PEIN GNSRNLSELF LQRNKISGQI TPTISSAYNL
Medtr1g069470 LVRVRIQNNF FSGTIPVGFG KLEKLQRLEL ANNSLTGGIP EDIAS---ST SLSFIDFSRN NLHSSLPSTI ISISNLQTFI VSENNLEGDI PDQFQDCPSL
Medtr7g098610 LVRVRIHNNF LSGKVPVGLG KLEKLQRLEL ANNSLTGEIP DDIPS---SM SLSFIDLSRN KLHSFLPSTI LSIPNLQVFK VSNNNLEGKI PGQFQDSPSL
Medtr1g080440 LVRVRIQNNL ISGTIPVGFG SLLSLQRLEL AKNNFTGQIP IDITS---ST SLSFIDVSWN HLESSLPSEI LSIPTLQTFI ASHNNLGGTI PDEFQGCPSL
Medtr4g03675 LIRVRIQNNN LNGSIPQTLT MLPNLTYLDL SNNNFKGEIP QEFGS----- -LQYLNISGN SFESELPNSI WNSSNLQIFS ASFSKITGQI P-DFSDCKSI
BAM3         LWRFRLGQNF LTSKLPKGLI YLPNLSLLEL QNNFLTGEIP EEEAGNAQFS SLTQINLSNN RLSGPIPGSI RNLRSLQILL LGANRLSGQI PGEIGSLKSL
BAM1         LTRIRMGENF LNGSIPKGLF GLPKLTQVEL QDNYLSGELP VAGGV---SV NLGQISLSNN QLSGPLPPAI GNFTGVQKLL LDGNKFQGPI PSEVGKLQQL
BAM2         LTRIRMGENF LNGSIPKELF GLPKLSQVEL QDNYLTGELP ISGGG--VSG DLGQISLSNN QLSGSLPAAI GNLSGVQKLL LDGNKFSGSI PPEIGRLQQL
CLV1         LTKIRIVKNL LNGTVPAGLF NLPLVTIIEL TDNFFSGELP VTMSG----D VLDQIYLSNN WFSGEIPPAI GNFPNLQTLF LDRNRFRGNI PREIFELKHL
PXY          LWRFRSQNNR LNGTIPIGFG SLRNLTFVDL SNNRFTDQIP ADFAT---AP VLQYLNLSTN FFHRKLPENI WKAPNLQIFS ASFSNLIGEI PNYVG-CKSF
AT4G28650    LVRVRMQNNL LNGSIPIGFG KLEKLQRLEL AGNRLSGGIP GDISD---SV SLSFIDFSRN QIRSSLPSTI LSIHNLQAFL VADNFISGEV PDQFQDCPSL
AT1G08590    LVRVRIQKNH ISGSIPAGSG DLPMLQHLEL AKNNLTGKIP DDIAL---ST SLSFIDISFN HLS-SLSSSI FSSPNLQTFI ASHNNFAGKI PNQIQDRPSL
AT3G24240    LVRLRLGFNR ITGEIPSGIG SLKKINFLDF SSNRLHGKVP DEIGS---CS ELQMIDLSNN SLEGSLPNPV SSLSGLQVLD VSANQFSGKI PASLGRLVSL
HSL1         LTRIRLAYNR FSGSVPTGFW GLPHVNLLEL VNNSFSGEIS KSIGG---AS NLSLLILSNN EFTGSLPEEI GSLDNLNQLS ASGNKFSGSL PDSLMSLGEL
AT5G48940    LVRLRLVNNR ITGEIPKGIG FLQNLSFLDL SENNLSGPVP LEISN---CR QLQMLNLSNN TLQGYLPLSL SSLTKLQVLD VSSNDLTGKI PDSLGHLISL
AT2G25790    LERVRLQNNG FSGKLPRGFT KLQLVNFLDL SNNNLQGNIN TWD-----MP QLEMLDLSVN KFFGELPD-F SRSKRLKKLD LSRNKISGVV PQGLMTFPEI
```

```
               801
MtNRLK1        LRLDMSFNNF SGTIPIEIGK CSSLTFLDLS QNKLSGPIPI QVSQIHILNY -LNVSWNYLN QTLPKELGSI KGLT------ ---------- -------SAD
Medtr3g44930   LKLDISSNNF SGTIPSEIGK CTLLTYLDLS QNQFSGPIPI QLAQIHILNH -LNVSWNHLN QSIPKELGAL KGLT------ ---------- -------SAD
Medtr2g005810  LKLDLTRNSL SGDIPPEIGY CVHLTYLDMS QNNLSGSIPP LISNIRILNY -LNLSRNHLN QSIPRSIGTM KSLT------ ---------- -------VAD
Medtr5g014700  SKIDFSHNRF SGPIAPEISK CKLLTFVDLS RNELSGIIPN EITHMKILNY -FNISRNHLV GSIPGSIASM QSLT------ ---------- -------SVD
Medtr4g097880  SKIDFSHNKF SGPIAPEISH CKLLTFVDLS RNELSGEIPK EITKMKILNY -LNLSRNHLV GTIPGSIASM QSLT------ ---------- -------SVD
SUNN           TRINISGNNL TGGIPKTVTQ CSSLTAVDFS RNMLTGEVPK GMKNLKVLSI -FNVSHNSIS GKIPDEIRFM TSLT------ ---------- -------TLD
CRA2           VKIDFSYNFL SGPIPSEIGN LRKLNLLMLQ ANKLNSSIPD SFSSLESLNL -LDLSSNLLT GNIPESLSVL LP-N------ ---------- -------SIN
Medtr1g069470  GVLDLSSNFF SGVIPESIAS CQKLVKLSLQ NNLLTGGIPK AIASMPTLSI -LDLANNSLT GQIPNNFGMS PALE------ ---------- -------TFN
Medtr7g098610  TVLDLSSNHL SGTIPDSIGS CQKLVNLNLQ NNLLIGEIPK ALANMPTMAM -LDLSNNSLT GHIPENFGVS PALE------ ---------- -------AFD
Medtr1g080440  SVLDLSNAYI SSPIPKGIAS CQKLVNLNLR NNHLTGEIPK SITNMPTLSV -LDLSNNSLT GRIPENFGSS PALE------ ---------- -------TMN
Medtr4g03675   YKIELQGNSI TGTIPWNIGD CEKLLQLNLS KNNLTGIIPY EISTLPSITD -VDLSQNSLT GTIPSSFNNC STLE------ ---------- -------NFN
BAM3           LKIDMSRNNF SGKFPPEFGD CMSLTYLDLS HNQISGQIPV QISQIRILNY -LNVSWNSFN QSLPNELGYM KSLT------ ---------- -------SAD
BAM1           SKIDFSHNLF SGRIAPEISR CKLLTFVDLS RNELSGEIPN EITAMKILNY -LNLSRNHLV GSIPGSISSM QSLT------ ---------- -------SLD
BAM2           SKLDFSHNLF SGRIAPEISR CKLLTFVDLS RNELSGDIPN ELTGMKILNY -LNLSRNHLV GSIPVTIASM QSLT------ ---------- -------SVD
CLV1           SRINTSANNI TGGIPDSISR CSTLISVDLS RNRINGEIPK GINNVKNLGT -LNISGNQLT GSIPTGIGNM TSLT------ ---------- -------TLD
PXY            YRIELQGNSL NGTIPWDIGH CEKLLCLNLS QNHLNGIIPW EISTLPSIAD -VDLSHNLLT GTIPSDFGSS KTIT------ ---------- -------TFN
AT4G28650      SNLDLSSNTL TGTIPSSIAS CEKLVSLNLR NNNLTGEIPR QITTMSALAV -LDLSNNSLT GVLPESIGTS PALE------ ---------- -------LLN
AT1G08590      SVLDLSFNHF SGGIPERIAS FEKLVSLNLK SNQLVGEIPK ALAGMHMLAV -LDLSNNSLT GNIPADLGAS PTLE------ ---------- -------MLN
AT3G24240      NKLILSKNLF SGSIPTSLGM CSGLQLLDLG SNELSGEIPS ELGDIENLEI ALNLSSNRLT GKIPSKIASL NKLSILDLSH NMLEGDLAPL ANIENLVSLN
HSL1           GTLDLHGNQF SGELTSGIKS WKKLNELNLA DNEFTGKIPD EIGSLSVLNY -LDLSGNMFS GKIPVSLQSL K-LN------ ---------- -------QLN
AT5G48940      NRLILSKNSF NGEIPSSLGH CTNLQLLDLS SNNISGTIPE ELFDIQDLDI ALNLSWNSLD GFIPERISAL NRLSVLDISH NMLSGDLSAL SGLENLVSLN
AT2G25790      MDLDLSENEI TGVIPRELSS CKNLVNLDLS HNNFTGEIPS SFAEFQVLSD -LDLSCNQLS GEIPKNLGNI ESLV------ ---------- -------QVN

               701
MtNRLK1        FSHNDFSGSV PEIGQFSVFN STSFVGNPKL CGY---DL-N PCNKS---SS ETLESQKN-G GEKPGIPAKY --KLLFALAL LVCSLVFATF AIMKGRKGI-
Medtr3g44930   FSHNNFSGSI PEGGQFSTFK ANSFEGNPQL CGYVLVEF-N PCKVS---ST DELESQQK-N GSRNGFPGKF --KLLFALAL LLCSLVFVTL AIMKSRKSR-
Medtr2g005810  FSFNEFSGKL PESGQFSFFN ATSFAGNPKL CGS--LLN-N PCKLT----- ------RM-K STPGKNNSDF --KLIFALGL LMCSLVFAVA AIIKAKSFK-
Medtr5g014700  FSYNNLSGLV PGTGQFSYFN YTSFLGNPDL CGP---YL-G ACKDG----- -VLDGPNQLH HVKGHLSSTV --KLLLVIGL LACSIVFAIA AIIKARSLK-
Medtr4g097880  FSYNNLTGLV PGTGQFSYFN YTSFLGNPEL CGP---YL-G PCKDG----- -VANGPRQ-P HVKGPLSSTV --KLLLVVGL LVCSAIFAVV TIFKARSLK-
SUNN           LSYNNFTGIV PTGGQFLVFN DRSFAGNPSL CFP---HQ-T TCSSL----- ---------- LYRSRKSHAK --EKAVVIAI VFATAVLMVI VTLHMMRKR-
CRA2           FSHNLLSGPI PPK-LIKGGL VESFAGNPGL C-----VM-M PVNAN----- -SSDQRNFPL CSHGYKSKKM --NTIWVAGV SVILIFVGAA LFLKKRCGKN
Medtr1g069470  VSYNKLEGPV PENGMLRAIN PNDLVGNAGL CGG---FF-P PCAKT----- -----SAYTM RHGSSHTKHI --IVGWIIGI SSILAIGVAA LVARSIYMKW
Medtr7g098610  VSYNKLEGSV PENGMLRTIN PNNLVGNAGL CGG---TL-L SCNQN----- -----SAYSS MHGSSHEKHI --ITGWIIGI SSILAIGITI LVARSLYVRW
Medtr1g080440  LSYNKLEGPV PSNGILLTMN PNDFVGNAGL CGS---IL-P PCSQS----- --STVTSQ-- -KRSSHISHI --VIGFVTGI SVILSLAAVY FGGKWLYNKC
Medtr4g03675   ISFNSLTGAI PSSGVFQSLH PSSYSGNENL CGV---LLAK PCADE----- -AVTSGENEL QVHRQQPKKT AGAIVWIIAA AFGIGLFVLV AGTRCFQTNY
BAM3           FSHNNFSGSV PTSGQFSYFN NTSFLGNPFL CGF---SS-N PCNGSQNQSQ SQLLNQNN-A RSRGEISAKF --KLFFGLGL LGFFLVFVVL AVVKNRRMR-
BAM1           FSYNNLSGLV PGTGQFSYFN YTSFLGNPDL CGP---YL-G PCKDG----- -VAKGGHQ-S HSKGPLSASM --KLLLVLGL LVCSIAFAVV AIIKARSLK-
BAM2           FSYNNLSGLV PSTGQFSYFN YTSFVGNSHL CGP---YL-G PC-------- --GKGTHQ-S HVK-PLSATT --KLLLVLGL LFCSMVFAIV AIIKARSLR-
CLV1           LSFNDLSGRV PLGGQFLVFN ETSFAGNTYL CLP---HR-V SCP------- -TRPGQTS-D HNHTALFSPS --RIVITVIA AITGLILISV AIRQMNKKK-
PXY            VSYNQLIGPI P-SGSFAHLN PSFFSSNEGL CGD---LVGK PCNSD---RF NAGNADID-G HHKEERPKKT AGAIVWILAA AIGVGFFVLV AATRCFQKSY
AT4G28650      VSYNKLTGPV PINGFLKTIN PDDLRGNSGL CGG---VL-P PCSKF----- -----QRATS SHSSLHGKRI --VAGWLIGI ASVLALGILT IVTRTLYKKW
AT1G08590      VSFNKLDGPI PSNMLFAAID PKDLVGNNGL CGG---VL-P PCSKS----- --LALSAKGR NPGRIHVNHA --VFGFIVGT SVIVAMGMMF LAGRWIYTRW
AT3G24240      ISYNSFSGYL PDNKLFRQLS PQDLEGNKKL CSS---TQ-D SCFLT----Y RKGNGLGDDG DASRTRKLRL --TLALLITL TVVLMILGAV AVIRARRNID
HSL1           LSYNRLSGDL PPS-LAKDMY KNSFIGNPGL CGD---IK-G LCGSE----- ---------- ----NEAKKR --GYVWLLRS IFVLAAMVLL AGVAWFYFKY
AT5G48940      ISHNRFSGYL PDSKVFRQLI GAEMEGNNGL CSK---GF-R SCFVS----- NSSQLTTQ-R GV-HSHRLRI --AIGLLISV TAVLAVLGVL AVIRAKQMIR
AT2G25790      ISHNLLHGSL PFTGAFLAIN ATAVEGNIDL CSENSASGLR PCKVV----- -RKRSTKSWW LIITSTFAAF --LAVLVSGF FIVLVFQRTH NVLEVKKV--
```

```
           601
MtNRLK1       ---------- ----K-RDSN PWKLTAFQKI E---YGSEDI LGCVKES-NI IGRGGAGVVY GGT-MPNGE- KVAVKKLLG- ---INKGCSY ----------
Medtr3g44930  ---------- ----R-NHSS SWKLTAFQKM E---YGSEEI IGCIKES-NV IGRGGAGVVY KGT-MPNGD- EIAVKKLLG- ---INKGNSS SHA-------
Medtr2g005810 ---------- ----K-KGPG SWKMTAFKKL E---FTVSDI LECVKDG-NV IGRGGAGIVY HGK-MPNGM- EIAVKKLLG- ---FGANNH- ----------
Medtr5g014700 ---------- ----KASEAR AWKLTSFQRL E---FTADDV LDSLKED-NI IGKGGAGIVY KGA-MPNGE- LVAVKRLPV- ---MSRGSSH ----------
Medtr4g097880 ---------- ----KASEAR AWKLTAFQRL D---FTVDDV LDSLKED-NI IGKGGAGIVY KGA-MPNGD- LVAVKRLPA- ---MSRGSSH ----------
SUNN          ---------- ----KRHMAK AWKLTAFQKL E---FRAEEV VECLKEE-NI IGKGGAGIVY RGS-MANGT- DVAIKRLVG- ---QGSGRN- ----------
CRA2          --VSAVEHEY T---LSSSFF SYDVKSFHMI S---FDQREI VESLVDK-NI MGHGGSGTVY KIE-LKTGD- VVAVKRLWS- ---RSSKDSS PEDALFV---
Medtr1g069470 --YTEGLCFR GRFYGGRKGW PWRLMAFQRL D---FTSTDI LSCIKET-NV IGMGGTGVVY KAE-IAQSST VVAVKKLWRT ESDIEVGSG- ----------
Medtr7g098610 --YTGGFCFR ERFYKGSKGW PWRLMAFQRL G---FTSTDI LACIKET-NV IGMGGTGIVY KAE-VPHSNT VVAVKKLWRS GNDVEVGRG- ----------
Medtr1g080440 --YMYNSFIY DWFKHNNEDW PWRLVAFQRI S---FTSSEI LTCIKES-NV IGMGGAGIVY KAE-IHKPQI TVAVKKLWR- ---SSPDIEN ----------
Medtr4g03675  NRRFNGND-- ----ANGEVG PWKLTAFQRL N---FTAEDV LECVSMSDKI LGMGSTGTVY KAE-LPGGE- IIAVKKLWS- ---KQKENST IIRR------
BAM3          ---------- ----K-NNPN LWKLIGFQKL G---FRSEHI LECVKEN-HV IGKGGRGIVY KGV-MPNGE- EVAVKKLLT- ---ITKGSSH ----------
BAM1          ---------- ----KASESR AWRLTAFQRL D---FTCDDV LDSLKED-NI IGKGGAGIVY KGV-MPNGD- LVAVKRLAA- ---MSRGSSH ----------
BAM2          ---------- ----NASEAK AWRLTAFQRL D---FTCDDV LDSLKED-NI IGKGGAGIVY KGT-MPKGD- LVAVKRLAT- ---MSHGSSH ----------
CLV1          ---------- ----N-QKSL AWKLTAFQKL D---FKSEDV LECLKEE-NI IGKGGAGIVY RGS-MPNNV- DVAIKRLVG- ---RGTGRS- ----------
PXY           GNRVDGGGR- ----NGGDIG PWKLTAFQRL N---FTADDV VECLSKTDNI LGMGSTGTVY KAE-MPNGE- IIAVKKLWG- ---KNKENGK IRRR------
AT4G28650     --YSNGFCGD ET--ASKGEW PWRLMAFHRL G---FTASDI LACIKES-NM IGMGATGIVY KAE-MSRSST VLAVKKLWRS AADIEDGTT- ----------
AT1G08590     DLYSNFAREY IFCKKPREEW PWRLVAFQRL C---FTAGDI LSHIKES-NI IGMGAIGIVY KAEVMRRPLL TVAVKKLWR- ---SPSPQND IEDHHQEEDE
AT3G24240     NERDS----- ----ELGETY KWQFTPFQKL N---FSVDQI IRCLVEP-NV IGKGCSGVVY RAD-VDNGE- VIAVKKLWP- -AMVNGGHDE KTKNV-----
HSL1          --RTFKKA-- ----RAMERS KWTLMSFHKL G---FSEHEI LESLDED-NV IGAGASGKVY KVV-LTNGE- TVAVKRLWT- GSVKETGDCD PEKGYKPGVQ
AT5G48940     --DDNDSE-- ----TGENLW TWQFTPFQKL N---FTVEHV LKCLVEG-NV IGKGCSGIVY KAE-MPNRE- VIAVKKLWP- -VTVPNLNEK TKSSGV----
AT2G25790     ---------- ----EQEDGT KWETQFFDSK FMKSFTVNTI LSSLKDQNVL VDKNGVHFV- ---------- VKEVKKYDS- ---------- ----------

           901
MtNRLK1        DNGLSAEIKT LGRIRHRYIV KLLAFCSNRD TNLLVYEYMT NGSLGEVLHG KRG-----GF LEWDVRVKIA TEAAKGLCYL HHDCCPLIVH RDVKSNNILL
Medtr3g44930   DNGFSAEIKT LGRIRHRYIV RLVAFCTNKE TNLLVYDYME NGSLGEVLHG KRG-----EF LKWNVRLKIA VEAAKGLCYL HHDCSPLIIH RDVKSNNILL
Medtr2g005810  DHGFRAEIQT LGNIRHRNIV RLLAFCSNKE TNLLVYEYMR NGSLGETLHG KKG-----AF LSWNFRYKIS IDSAKGLCYL HHDCSPLILH RDVKSNNILL
Medtr5g014700  DHGFNAEIQT LGRIRHRHIV RLLGFCSNHE TNLLVYEYMP NGSLGEVLHG KKG-----GH LYWDTRYKIA VEAAKGLCYL HHDCSPLIVH RDVKSNNILL
Medtr4g097880  DHGFNAEIQT LGRIRHRHIV RLLGFCSNHE TNLLVYEYMP NGSLGEVLHG KKG-----GH LHWDTRYKIA VEAAKGLCYL HHDCSPLIVH RDVKSNNILL
SUNN           DYGFKAEIET LGRIRHRNIM RLLGYVSNKD TNLLLYEYMP NGSLGEWLHG AKG-----CH LSWEMRYKIA VEAAKGLCYL HHDCSPLIIH RDVKSNNILL
CRA2           DKALKAEVET LGSIRHKNIV KLYCCFSSLD CSLLVYEYMP NGTLYDSLH- -KG----WIH LDWPTRYRIA LGIAQGVAYL HHDLVFPIIH RDIKSTNILL
Medtr1g069470  -DDLVGEVNL LGRLRHRNIV RLLGFLYNDT DVMIVYEFMV NGNLGDAMHG KQS--E-RLL VDWVSRYNIA LGIAQGLAYL HHDCHPPVIH RDIKSNNILL
Medtr7g098610  SDELVGEVNL LGRLRHRNIV RLLGFLHNDT DLMIVYEFMN NGNLGDALHG RQS--V-RHL VDWVSRYNIA LGVAQGLAYL HHDCHPPVIH RDIKSNNILL
Medtr1g080440  GNDVLREVEL LGRLRHRNIV RLLGYVHNER DVIMVYEYMI NGNLGTALHG EQS--A-RLL VDWVSRYNIA LGVAQGMNYL HHDCHPPVIH RDIKSNNILL
Medtr4g03675   RRGVLAEVDV LGNVRHRNIV RLLGCCSNKE ITMLLYEYMP NGNLDEFLHA KNKGDNMVIV SDWFTRYKIA LGVAQGISYL HHDCDPVIVH RDLKPSNILL
BAM3           DNGLAAEIQT LGRIRHRNIV RLLAFCSNKD VNLLVYEYMP NGSLGEVLHG KAG-----VF LKWETRLQIA LEAAKGLCYL HHDCSPLIIH RDVKSNNILL
BAM1           DHGFNAEIQT LGRIRHRHIV RLLGFCSNHE TNLLVYEYMP NGSLGEVLHG KKG-----GH LHWDTRYKIA LEAAKGLCYL HHDCSPLIVH RDVKSNNILL
BAM2           DHGFNAEIQT LGRIRHRHIV RLLGFCSNHE TNLLVYEYMP NGSLGEVLHG KKG-----GH LHWNTRYKIA LEAAKGLCYL HHDCSPLIVH RDVKSNNILL
CLV1           DHGFTAEIQT LGRIRHRHIV RLLGYVANKD TNLLLYEYMP NGSLGELLHG SKG-----GH LQWETRHRVA VEAAKGLCYL HHDCSPLILH RDVKSNNILL
PXY            KSGVLAEVDV LGNVRHRNIV RLLGCCTNRD CTMLLYEYMP NGSLDDLLHG GDK--TMTAA AEWTALYQIA IGVAQGICYL HHDCDPVIVH RDLKPSNILL
AT4G28650      -GDFVGEVNL LGKLRHRNIV RLLGFLYNDK NMMIVYEFML NGNLGDAIHG KNA--AGRLL VDWVSRYNIA LGVAHGLAYL HHDCHPPVIH RDIKSNNILL
AT1G08590      EDDILREVNL LGGLRHRNIV KILGYVHNER EVMMVYEYMP NGNLGTALHS KDE--K-FLL RDWLSRYNVA VGVVQGLNYL HNDCYPPIIH RDIKSNNILL
AT3G24240      RDSFSAEVKT LGTIRHKNIV RFLGCCWNRN TRLLMYDYMP NGSLGSLLHE RRG-----SS LDWDLRYRIL LGAAQGLAYL HHDCLPPIVH RDIKANNILI
HSL1           DEAFEAEVET LGKIRHKNIV KLWCCCSTRD CKLLVYEYMP NGSLGDLLHS SKG-----GM LGWQTRFKII LDAAEGLSYL HHDSVPPIVH RDIKSNNILI
AT5G48940      RDSFSAEVKT LGSIRHKNIV RFLGCCWNKN TRLLMYDYMS NGSLGSLLHE RSG----VCS LGWEVRYKII LGAAQGLAYL HHDCVPPIVH RDIKANNILI
AT2G25790      LPEMISDMRK LSD--HKNIL KIVATCRSET VAYLIHEDVE GKRLSQVLSG ---------- LSWERRRKIM KGIVEALRFL HCRCSPAVVA GNLSPENIVI
```

```
               1201
MtNRLK1        NSEFEAHVAD FGLAKFLLQD TGGTSECMSS IVGSYGYIAP EYAYTLKVDE KSDVYSFGVV LLELLTGRRP VG-DFGEEGM D--IVQWTKL KTD-----WN
Medtr3g44930   NSEFEAHVAD FGLAKFLQDN --GNSECMSS IAGSYGYIAP EYAYTLKVDE KSDVYSFGVV LLELITGKRP VG-DFEEEGL D--IVQWTKM KTN-----WN
Medtr2g005810  SSNFEAHVAD FGLAKFLVDG --AAAECMSS IAGSYGYIAP EYAYTLRVDE KSDVYSFGVV LLELLTGRKP VG-DFG-EGV D--LVQWCKK ATN-----GR
Medtr5g014700  DSNYEAHVAD FGLAKFLQDS --GTSECMSA IAGSYGYIAP EYAYTLKVDE KSDVYSFGVV LLELVTGRKP VG-EFG-DGV D--IVQWVRK MTD-----SN
Medtr4g097880  DSGFEAHVAD FGLAKFLQDS --GTSECMSA IAGSYGYIAP EYAYTLKVDE KSDVYSFGVV LLELVAGRKP VG-EFG-DGV D--IVQWVRK MTD-----SN
SUNN           DADFEAHVAD FGLAKFLYDP --GASQSMSS IAGSYGYIAP EYAYTLKVDE KSDVYSFGVV LLELIIGRKP VG-EFG-DGV D--IVGWINK TELELYQPSD
CRA2           DEDYHPKVAD FGIAKVLQAR -GAKDSTTTV IAGTYGYLAP EYAYSPRATT KCDVYSFGVI LLELLTGRKP IESEFG-ENR N--IVFWVAN KVEG----KE
Medtr1g069470  DANLEARIAD FGLAKMMVRK ----NETVSM IAGSYGYIAP EYGYSLKVDE KIDIYSFGIV LLELITGKRP IDPDFG-ESV D--IVGWIRR KID-------
Medtr7g098610  DANLEARIAD FGLAKMMIQK ----NETVSM VAGSYGYIAP EYGYALKVDE KIDVYSYGVV LLELVTGKRP LDSEFG-ESV D--IVEWIRR KIR------E
Medtr1g080440  DANLEARIAD FGLARMMIQK ----NETVTM VAGSYGYIAP EYGYTLKVDE KIDIYSYGVV LLELLTGKMP LDHTFE-EAV D--IVEWIQK KRN-------
Medtr4g03675   DGEMEARVAD FGVAKLIQTD -----ESMSV IAGSYGYIAP EYAYTLQVDE KSDIYSYGVV LMEILSGKRS VDQEFG-DGN S--IVDWVKS KIK------S
BAM3           GPEFEAHVAD FGLAKFMMQD -NGASECMSS IAGSYGYIAP EYAYTLRIDE KSDVYSFGVV LLELITGRKP VD-NFGEEGI D--IVQWSKI QTN-----CN
BAM1           DSNFEAHVAD FGLAKFLQDS --GTSECMSA IAGSYGYIAP EYAYTLKVDE KSDVYSFGVV LLELVTGRKP VG-EFG-DGV D--IVQWVRK MTD-----SN
BAM2           DSNFEAHVAD FGLAKFLQDS --GTSECMSA IAGSYGYIAP EYAYTLKVDE KSDVYSFGVV LLELITGKKP VG-EFG-DGV D--IVQWVRS MTD-----SN
CLV1           DSDFEAHVAD FGLAKFLVDG --AASECMSS IAGSYGYIAP EYAYTLKVDE KSDVYSFGVV LLELIAGKKP VG-EFG-EGV D--IVRWVRN TEEEITQPSD
PXY            DADFEARVAD FGVAKLIQTD -----ESMSV VAGSYGYIAP EYAYTLQVDK KSDIYSYGVI LLEIITGKRS VEPEFG-EGN S--IVDWVRS KLK------T
AT4G28650      DANLDARIAD FGLARMMARK ----KETVSM VAGSYGYIAP EYGYTLKVDE KIDIYSYGVV LLELLTGRRP LEPEFG-ESV D--IVEWVRR KIR------D
AT1G08590      DSNLEARIAD FGLAKMMLHK ----NETVSM VAGSYGYIAP EYGYTLKIDE KSDIYSLGVV LLELVTGKMP IDPSFE-DSI D--VVEWIRR KVK------K
AT3G24240      GLDFEPYIAD FGLAKLVDEG --DIGRCSNT VAGSYGYIAP EYGYSMKITE KSDVYSYGVV VLEVLTGKQP IDPTVP-EGI H--LVDWVRQ ----------
HSL1           DGDYGARVAD FGVAKAVDLT -GKAPKSMSV IAGSCGYIAP EYAYTLRVNE KSDIYSFGVV ILEIVTRKRP VDPELG--EK D--LVKWVCS TLD-------
AT5G48940      GPDFEPYIGD FGLAKLVDDG --DFARSSNT IAGSYGYIAP EYGYSMKITE KSDVYSYGVV VLEVLTGKQP IDPTIP-DGL H--IVDWVKK IRD-------
AT2G25790      DVTDEPRLC- LGLPGLL--- ------CMDA -----AYMAP ETREHKEMTS KSDIYGFGIL LLHLLTGKCS SSNEDIESGV NGSLVKWARY SYS-------

               1101
MtNRLK1        KESVVKILDG RL--HNNIPL DEAMQLFFVA MCCVEEQSVE RPTMREVVEM LGQVKQPNIF QV-------- ---------- ---------- ----------
Medtr3g44930   KDMVMKILDE RL--P-QIPL HEAKQVFFVA MLCVHEHSVE RPTMREVVEM LAQAK----- ---------- ---------- ---------- ----------
Medtr2g005810  REEVVNIIDS RL--M-VVPK EEAMHMFFIA MLCLEENSVQ RPTMREVVQM LSEFPRQSTS SSSSS----- ---------- SSSSSSNSSS NPPIKKLIQN
Medtr5g014700  KEGVLKVLDP RL--S-SVPL QEVMHVFYVA ILCVEEQAVE RPTMREVVQI LTELPKSTES ---------- ---------- KLGDSTITES SLSSSNALES
Medtr4g097880  KEGVLKVLDP RL--P-SVPL NEVMHVFYVA MLCVEEQAVE RPTMREVVQM LTELPKPPSS KHVEE----- ---------- DLTTLTINES SLSSSNSLES
SUNN           KALVSAVVDP RL--N-GYPL TSVIYMFNIA MMCVKEMGPA RPTMREVVHM LTNPPHSTSH N--------- ---------- ---------- ----------
CRA2           GARPSEVFDP KL--SCSF-K DDMVKVLRIA IRCSYKAPAS RPTMKEVVQL LIEAEPRKSD SC-------- ---------- KLSTKDVSTN VTLVKKSFEL
Medtr1g069470  KNSPEEALDP SVG-NCKHVQ EEMLLVLRIA LLCTAKLPKE RPSMRDVIMM LGEAKPRRKG ---------- ---------- GKKNETLTAN KESM------
Medtr7g098610  NKSLEEALDP SVG-NCRHVI EEMLLVLRIA VVCTAKLPKE RPSMRDVIMM LGEAKPRRKI NGNNE----- ---------- TSLAANNNNK EMSVFST---
Medtr1g080440  NKAMLEALDP TIAGQCKHVQ EEMLLVLRIA LLCTAKLPKE RPSMRDIITM LGEAKPRRKS IC-------- ---------- GNGRQESSIE KGTIFTT---
Medtr4g03675   KDGIEGILDK NAGAGCNSVR EEMKQMLRIA LLCTSRNPAD RPSMRDVVLM LQAAKPKRKL F-DSV----- ---------- VVSHCASGDN VDGGGGDDIP
BAM3           RQGVVKIIDQ RL--S-NIPL AEAMELFFVA MLCVQEHSVE RPTMREVVQM ISQAKQPNTF ---------- ---------- ---------- ----------
BAM1           KDSVLKVLDP RL--S-SIPI HEVTHVFYVA MLCVEEQAVE RPTMREVVQI LTEIPKLPPS KD-------- ---------- QPMTESAPES ELSPKSGVQS
BAM2           KDCVLKVIDL RL--S-SVPV HEVTHVFYVA LLCVEEQAVE RPTMREVVQI LTEIPKIPLS KQ-------- ---------- QAAESDVTEK APAINESSPD
CLV1           AAIVVAIVDP RL--T-GYPL TSVIHVFKIA MMCVEEEAAA RPTMREVVHM LTNPPKSVAN LIAF------ ---------- ---------- ----------
PXY            KEDVEEVLDK SMGRSCSLIR EEMKQMLRIA LLCTSRSPTD RPPMRDVLLI LQEAKPKRKT VGDNV----- ---------- IVVGDVNDVN FEDVCSVDVG
AT4G28650      NISLEEALDP NVG-NCRYVQ EEMLLVLQIA LLCTTKLPKD RPSMRDVISM LGEAKPRRKS NSNE------ ---------- ENTSRSLAEK HSSVFST---
AT1G08590      NESLEEVIDA SIAGDCKHVI EEMLLALRIA LLCTAKLPKD RPSIRDVITM LAEAKPRRKS VC-------- ---------- QVAGDLPIFR NSPVVGLI--
AT3G24240      NRGSLEVLDS TLRSRTEAEA DEMMQVLGTA LLCVNSSPDE RPTMKDVAAM LKEIKQEREE YAKVDLLLKK SPPPTTTMQE ECRKNEMMMI PAAAASSSKE
HSL1           QKGIEHVIDP KL--D-SCFK EEISKILNVG LLCTSPLPIN RPSMRRVVKM LQEIGGGDED SLHKI----- ---------- RDDKDGKLTP YYNEDTSDQG
AT5G48940      ----IQVIDQ GLQARPESEV EEMMQTLGVA LLCINPIPED RPTMKDVAAM LSEICQEREE SMKVDGCSGS CNNGRERGKD DSTSSVMQQT AKYLRSSSTS
AT2G25790      NCHIDTWIDS SI--DTSVHQ REIVHVMNLA LKCTAIDPQE RPCTNNVLQA LESTSSSSSS CT-------- ---------- TYLSKILSLA ----------
```

```
               1001
MtNRLK1        ---------- ---------- ---------
Medtr3g44930   ---------- ---------- ---------
Medtr2g005810  HKLPCPPT-- -----FKQDL LV-------
Medtr5g014700  PTAASKDHQH PPQSPPPDLL SI-------
Medtr4g097880  PSKD------ -----PKDLL SI-------
SUNN           ---------- --------LI NL-------
CRA2           ---------- ---------- ---------
Medtr1g069470  ---------- ---------- ---------
Medtr7g098610  ---------- -----SPVSG LL-------
Medtr1g080440  ---------- -----SPVAS LL-------
Medtr4g03675   LAQ------- -----KPITN ES-------
BAM3           ---------- ---------- ---------
BAM1           ---------- -----PPDLL NL-------
BAM2           SGS------- -----PPDLL SN-------
CLV1           ---------- ---------- ---------
PXY            HDV------- -----KCQRI GV-------
AT4G28650      ---------- -----SPVNG LL-------
AT1G08590      ---------- ---------- ---------
AT3G24240      MRREERLLKS NNTSFSASSL LYSSSSSIE
HSL1           SIA------- ---------- ---------
AT5G48940      FSASSLLYSS SSSATSNVRP NLK------
AT2G25790      ---------- ---------- ---------
```

Fig. S1. Protein sequence alignments used to generate a similarity tree, highlighting functional domains

Aligments were generated as described in the Methods to generate the similarity tree shown in Figure 1. Leucine-Rich Repeats (LRR), defined based on the LRRFinder software (http://www.lrrfinder.com/), are highlighted by blue bars, and the transmembrane domain, the kinase-like domain including the serine/threonine protein kinase active-site signature (green bar), defined using the Prosite motif database (http://www.genome.jp/tools/motif/), are highlighted respectively by a red bar and light-blue brackets.

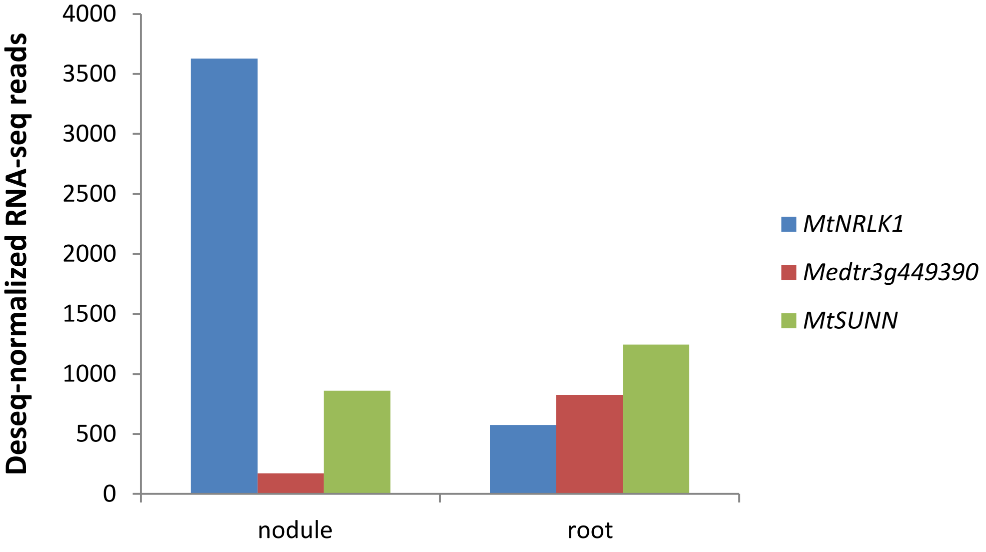

Fig. S2. Expression in roots and nodules of *MtNRLK1* and *MtSUNN*.
Absolute Deseq-normalized RNA-seq read numbers from root and nodule samples generated in Roux et al.[36] are shown for *MtNRLK1*, *Medtr3g449390*, and *MtSUNN*.

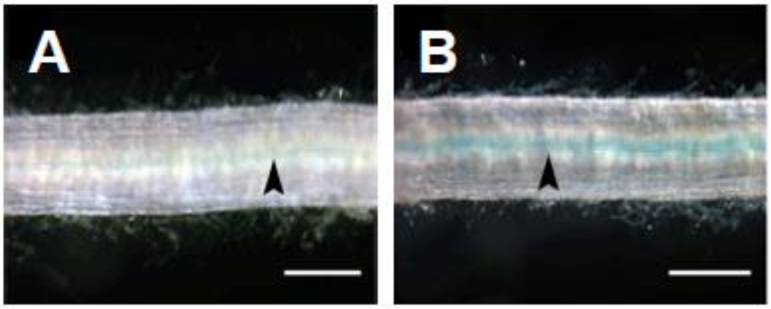

Fig. S3. Spatial expression of *MtNRLK1* and *MtSUNN* in uninoculated roots.
Promoter:GUS activity of *MtNRLK1* (A) and *MtSUNN* (B). A blue staining was observed in the root stele (arrowheads) in both cases. Bars = 0.5 mm.

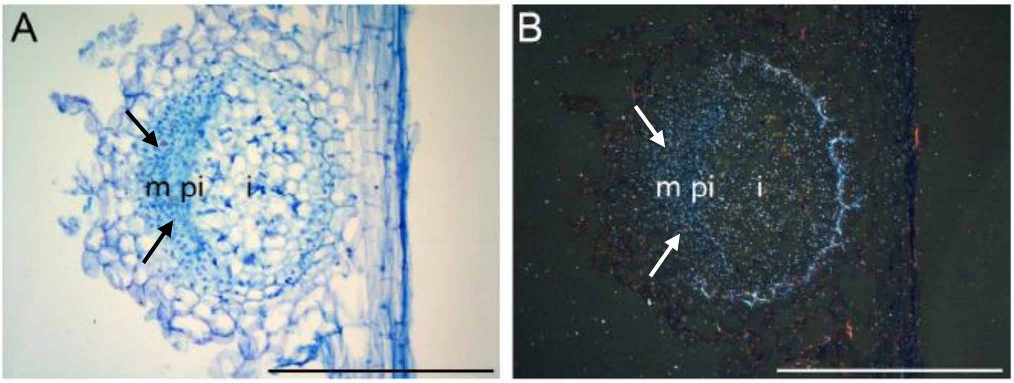

Fig. S4. ***MtNRLK1*** **expression in nodules detected by** ***in situ*** **hybridization.**
A-B, Microscopic images of *MtNRLK1 in situ* hybridizations on sections of a round-shaped nodule (12 days post *Rhizobium* inoculation). On the bright field picture (A), the *MtNRLK1* signal is seen as black spots (black arrows); on the dark field picture (B), the signal is detected as white spots (white arrows). *MtNRLK1* transcripts are mostly detected in the infection zone (inf), but also in the pre-infection zone (pi), in the proximal part of the meristem (m). Bars = 0,5 mm.

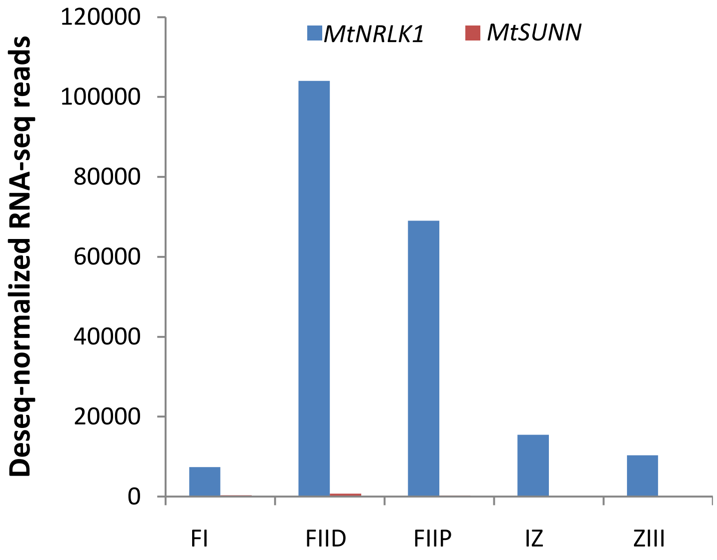

Fig. S5. Expression in roots and nodules of the *MtNRLK1* and *MtSUNN* genes.
Absolute Deseq-normalized RNA-seq read numbers from samples obtained by laser dissection of different nodule zones were retrieved from Roux et al.[36]. FI, Fraction I corresponding to the apical meristem; FIId, distal Fraction II corresponding to the distal part of the differentiation / rhizobial infection zone; FIIp, proximal Fraction II corresponding to the proximal part of the differentiation / rhizobial infection zone; IZ, Inter-Zone between FII and FIII; FIII, Fraction III corresponding to the nitrogen-fixing zone.

A

```
>Medtr5g090100.1
ATGGATACTCCTAGTTTCACCTTTGTACTATATACCCTCTTTCTTACTCTTTCTGTTTCT
GTTTCTTCATCATCATCACTTCCTATGTCTTTGAAAACACAAGCTTCTATCTTAGTTTCT
CTCAAACAGGATTTTGAATCCAAAACTTCTTTAAAAAGTTGGAATATCTCTAACTACATG
TCCCTTTGTACTACTTGGTATGGTATTCAATGTGACACAAACAATAGTTCTGTTGTGTCA
CTGGATATATCAAACTTGAATGTTTCTGGAACTTTTTCATCTTCCATCACAAAACTATCA
AACTTGAGATTCCTCAACATATCCAACAACATGTTCAATGGAAATCTAAGTTGGAAATTC
TCTCATTTGAAAGAGCTTGAAGTTCTTGATGCTTACAATAATGAGTTCAATTGTTCACTC
CCTCTTGGTGTCACTGAGTTACCTAAACTCAAGTACTTGAATTTTGGAGGAAATTTTTTC
TATGGAGAAATTCCTTCAAAATATGGTAACATGTTGCAGCTTAACTATTTGTCTCTTGCA
GGTAATGATTTGAGAGGTTTCATACCTTTTGAGCTTGGAAATCTTACTAATCTTACACAC
CTTTTGTTAGGTTATTACAATGAGTTTGATGGTGAAATTCCACCTCATTTTGGTAACCTT
GTTAATTTAGTTCATCTTGATCTTGCAAACTGTGGTTTGAAAGGTTCAATTCCACATGAG
CTAGGAAAACTTTACAAACTAGACACACTTTTCTTGCAAACCAATCAACTTAATGGTTCA
ATTCCACCTCAATTAGGGAATCTTAGTAGTTTGAAATCTCTTGATATGTCAAACAATGAG
TTAAATGGAAACATACCTAATGAGTTTTCAAATCTTCGCGAACTCACACTCTTGAACTTG
TTCATCAACAAACTCTATGGTGAAATTCCATCTTTTTTTTCTGAGTTACCAAATTTGGAA
GTGTTAAAGCTTTGGCAAAACAACTTCACAGGTTCTATTCCTTCTAAGCTTGGAAAGAAT
GGTAAATTGAGTGAACTTGATTTATCAACAAATAAACTCACTGGATTAGTACCTAAATCT
TTATGTCTTGGAAAAAGGTTAAAGATTCTAATCTTGCTTAACAATTTTTTGTTTGGTTCT
TTGCCTAATGAGTTTGGTCAATGTTACACACTTCAAAGAGTTCGTTTAGGACAAAACTAT
TTAACAGGTTCAATACCAAAGGGTTTTCTTTATTTGCCTCAACTTTCACTTCTTGAACTT
CAAAACAATTTGCTTGGTGGTTTTCTTCCACAACAAGAAATCACAAACACAAACACTTCT
AAACTTGGAGAGATAAATCTTTCGAACAACCGTTTATCCGGATCACTACCAAATTCTATT
GGAAACTTTCCAAACTTGCAGATTCTGCTTCTTCATGGAAATAGATTCTCAGGTGAAATC
CCTTCAGATATAGGAAAGTTGAAGAACATTCTCAGGTTGGATATGAGTTTCAACAACTTT
TCAGGAACTATTCCTATTGAAATAGGTAAATGTTCTTCACTTACCTTTTTAGATTTGAGT
CAGAACAAATTATCAGGTCCTATTCCAATTCAGGTTTCTCAAATTCACATACTTAACTAT
CTTAATGTCTCATGGAACTACCTAAACCAAACACTTCCAAAGGAACTTGGTTCTATTAAG
GGCTTAACTTCAGCTGATTTCTCTCATAATGACTTCTCTGGCTCAGTTCCTGAAATAGGA
CAATTCTCAGTGTTCAACTCTACATCCTTTGTTGGTAATCCAAAGTTATGTGGATATGAT
TTGAATCCATGCAACAAATCTTCATCAGAAACATTAGAGTCTCAGAAAAATGGTGGTGAG
AAACCTGGAATTCCTGCAAAGTATAAGCTTCTGTTTGCATTAGCACTTTTGGTTTGTTCA
TTAGTTTTTGCAACATTTGCTATCATGAAGGGTAGAAAAGGAATTAAGAGGGACTCAAAT
CCATGGAAGTTAACAGCATTTCAGAAGATTGAATATGGAAGTGAAGACATCTTAGGATGT
GTAAAGGAAAGTAACATCATAGGAAGAGGAGGTGCTGGTGTTGTATATGGAGGAACAATG
CCAAATGGAGAAAAAGTAGCTGTGAAAAAGTTATTAGGAATTAACAAAGGTTGTTCTTAT
GATAATGGTTTATCAGCTGAAATAAAAACACTTGGTAGAATAAGGCACAGATACATTGTG
AAGTTGTTAGCATTTTGTTCAAATAGAGACACTAATTTGCTTGTTTATGAGTACATGACA
AATGGTAGTTTAGGTGAAGTGTTGCATGGAAAAGAGGGGGGTTTCTTGAGTGGGATGTT
AGGGTGAAAATAGCAACTGAAGCTGCTAAAGGACTTTGCTATTTGCACCATGATTGTTGT
CCTTTGATTGTTCATAGAGATGTTAAGTCAAATAACATATTGTTGAACTCTGAGTTTGAG
GCTCATGTTGCTGATTTTGGACTTGCTAAGTTCTTGCTGCAAGATACTGGTGGGACTTCT
GAATGCATGTCTTCCATTGTTGGTTCTTATGGCTACATTGCTCCAGTACTTTTAATTTTG
ATATTCAAAATATAGATATATATTTAAATCAGTTAGACTGAAAATTGAAAACTATTTAAA
GTAGAAGTTATGGACACATTATTGCCTAGCTTAAGCTACCCGAAAATATTGTTTAGATTA
CTGTAGTGATTTGACCAGGTAGAATGAGTGAGTCCAAATCAACATTAGTTATGATTATTT
TGGATCCATTTAATAATCAGATAAATATTCATAGTCAAAAAGTCAATATGAAGGTAATGA
ATATACTTTTTTTCTGTTATTTTTAATTGACACAAAACATTCCCTTTTTACACCACTATA
AATAGCACATTCTTGCCATTATCTGGGACCACATTATGTCCTTCATGTACGTGAATTCCA
AGACTAGTAAATGCTCCACTTACAAAGGAAAATTCTTCAATCTTTTGCTTTTCTTATACC
AAGCTTGGTTTGTTCTTTTGCATGCCTTGAAACCATAAAATTATATTCAAACCCAAACAA
ATCACAACCATCTTTTTCTGACTATATATAACCCACTAATATATATCATATCTCTAGTAG
CTAACATATGCACAATAACCAAAAGCATATAGATATTATAGTTTAAATTGTGAAAGGAAG
CAATTATGTAAACTGATTCTGAATTATGTTATGTTACAGAATATGCATACACATTGAAAG
TGGATGAGAAAAGTGATGTATACAGCTTTGGGGTGGTGCTACTAGAACTTTTAACAGGGA
GAAGGCCAGTGGGAGATTTTGGTGAAGAAGGAATGGATATTGTTCAATGGACCAAGTTAA
AAACTGATTGGAACAAAGAAAGTGTGGTGAAGATTCTTGATGGGAGGTTACATAACAATA
TTCCTTTAGATGAAGCAATGCAGTTATTCTTTGTAGCTATGTGTTGTGTTGAGGAACAAA
GTGTGGAACGACCAACCATGAGAGAAGTGGTTGAAATGCTAGGACAAGTAAAGCAACCAA
ATATATTTCAAGTGTAA
```

C

```
MDTPSFTFVLYTLFLTLSVSVSSSSSLPMSLKTQASILVSLKQDFESKTSLKSWNISNYM
SLCTTWYGIQCDTNNSSVVSLDISNLNVSGTFSSSITKLSNLRFLNISNNMFNGNLSWKF
SHLKELEVLDAYNNEFNCSLPLGVTELPKLKYLNFGGNFFYGEIPSKYGNMLQLNYLSLA
GNDLRGFIPFELGNLTNLTHLLLGYYNEFDGEIPPHFGNLVNLVHLDLANCGLKGSIPHE
LGKLYKLDTLFLQTNQLNGSIPPQLGNLSSLKSLDMSNNELNGNIPNEFSNLRELTLLNL
FINKLYGEIPSFFSELPNLEVLKLWQNNFTGSIPSKLGKNGKLSELDLSTNKLTGLVPKS
LCLGKRLKILILLNNFLFGSLPNEFGQCYTLQRVRLGQNYLTGSIPKGFLYLPQLSLLEL
QNNLLGGFLPQQEITNTNTSKLGEINLSNNRLSGSLPNSIGNFPNLQILLLHGNRFSGEI
PSDIGKLKNILRLDMSFNNFSGTIPIEIGKCSSLTFLDLSQNKLSGPIPIQVSQIHILNY
LNVSWNYLNQTLPKELGSIKGLTSADFSHNDFSGSVPEIGQFSVFNSTSFVGNPKLCGYD
LNPCNKSSSETLESQKNGGEKPGIPAKYKLLFALALLVCSLVFATFAIMKGRKGIKRDSN
PWKLTAFQKIEYGSEDILGCVKESNIIGRGGAGVVYGGTMPNGEKVAVKKLLGINKGCSY
DNGLSAEIKTLGRIRHRYIVKLLAFCSNRDTNLLVYEYMTNGSLGEVLHGKRGGFLEWDV
RVKIATEAAKGLCYLHHDCCPLIVHRDVKSNNILLNSEFEAHVADFGLAKFLLQDTGGTS
ECMSSIVGSYGYIAPEYAYTLKVDEKSDVYSFGVVLLELLTGRRPVGDFGEEGMDIVQWT
KLKTDWNKESVVKILDGRLHNNIPLDEAMQLFFVAMCCVEEQSVERPTMREVVEMLGQVK
QPNIFQV
```

B

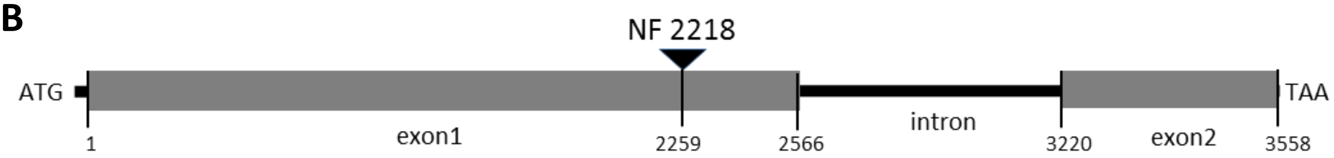

Fig. S6. MtNRLK1 genomic and protein sequences, highlighting regions selected for RNAi and the location of the *Tnt1* insertion in the *nrlk1* mutant.

Fig. S6. MtNRLK1 genomic and protein sequences, highlighting regions selected for RNAi and the location of the *Tnt1* insertion in the *nrlk1* mutant.

A. The nucleotide sequence corresponding to the *MtNRLK1* opening reading frame was retrieved from the *M. truncatula* Phytozome database (https://phytozome.jgi.doe.gov/pz/portal.html). Letters in grey correspond to the intron. Regions highlighted in green and yellow are respectively regions used for the RNAi constructs #1 and #2. The nucleotide highlighted in black indicates the location of the *Tnt1* insertion in the NF2218 mutant *nrlk1* line (http://bioinfo4.noble.org/mutant/).

B. Scheme of the *MtNRLK1* gene showing introns and exons as well as the *Tnt1* insertion location in the NF2218 mutant *nrlk1* line.

C. MtNRLK1 protein sequence. The amino acid highlighted in black corresponds to the region where the *Tnt1* insertion occurs in the NF2218 mutant genomic DNA. Leucine-Rich Repeats (LRR), defined based on the LRRFinder software (http://www.lrrfinder.com/), are highlighted in green, and the transmembrane domain, the kinase-like domain including the serine/threonine protein kinase active-site signature (in red), defined using the Prosite motif database (http://www.genome.jp/tools/motif/), are highlighted respectively in pink and blue.

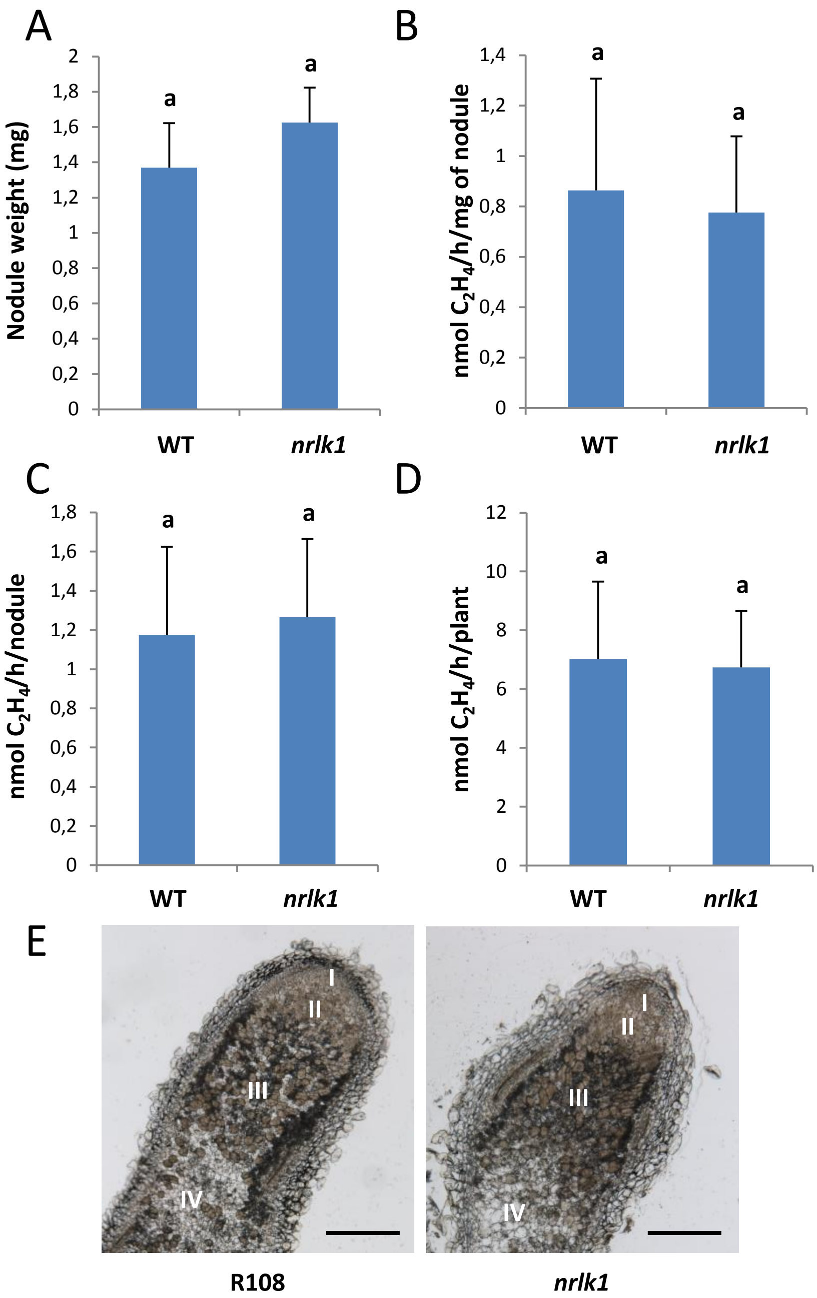

Fig. S7. *Mtnrlk1* mutants have no significant nitrogen fixation defect and a nodule patterning similar to the wild-type.

A. Nodule weight (mg) of Wild-Type (WT) and *nrlk1* mutant nodules 3 weeks after inoculation with the *S. medicae* WSM419 strain. B-D. Acetylene Reduction Assays (ARA) were conducted on the same plants as in (A), and nmol of C_2H_4 produced were normalized per hour and per nodule weight (mg; B), per nodule (C), or per plant (mg; D). In A-D, error bars represent standard deviations (n>11) and a Mann and Whitney test was performed to assess significant differences (α< 0,05). E. Histological section of a representative Wild-Type (WT) or *nrlk1* mutant nodule from the same plants as in A-D. Nodules zones (I to IV are defined according to Vasse et al. (1990). Bars = 500 µm.

Vasse J, de Billy F, Camut S, Truchet G. Correlation between ultrastructural differentiation of bacteroids and nitrogen fixation in alfalfa nodules. *J Bacteriol.* **172,** 4295-306 (1990).

Table S1. Primers used in this study.

Gene	Forward primer (5’-> 3’)	Reverse primer (5’-> 3’)
Gateway cloning		
MtNRLK1 promoter	ATTATGCACCCAACACGAAA	CATTATTTGTGGAATAACTAGC
MtSUNN promoter	TCCCTTTCACACACAAAATGA	CATCTTTCTCTTTTGCTCAGTTTT
qRT-PCR amplification		
MtNRLK1	GCCAGTGGGAGATTTT	GGTCGTTCCACACTTTGTTCC
MtSUNN	GCTCCTACGGCTACATTGC	ACTGGCTTCCTTCCTATTATCAG
40S Ribosomal Protein	GCCATTGTCCAAGTTGATGCTG	TTTTCCTACCAACTTCAACACCG
Histone 3-Like	ATTCCAAAGGCGGCTGCATA	CTTTGCTTGGTGCTGTTTAGATGG
**In situ *hybridization***		
MtNRLK1	GCCAGTGGGAGATTTTGGTGAAG	AAGCCAAGCCAAGGTATGC
